# Genome-Wide Association Studies of 11 Agronomic Traits in Cassava (*Manihot esculenta* Crantz)

**DOI:** 10.3389/fpls.2018.00503

**Published:** 2018-04-19

**Authors:** Shengkui Zhang, Xin Chen, Cheng Lu, Jianqiu Ye, Meiling Zou, Kundian Lu, Subin Feng, Jinli Pei, Chen Liu, Xincheng Zhou, Ping’an Ma, Zhaogui Li, Cuijuan Liu, Qi Liao, Zhiqiang Xia, Wenquan Wang

**Affiliations:** ^1^College of Plant Science and Technology, Huazhong Agricultural University, Wuhan, China; ^2^Institute of Tropical Bioscience and Biotechnology, Chinese Academy of Tropical Agricultural Sciences, Haikou, China; ^3^Tropical Crops Genetic Resources Institute, Chinese Academy of Tropical Agricultural Sciences, Danzhou, China; ^4^Wuming Agricultural Technology Extension Center, Nanning, China; ^5^Hepu Institute of Agricultural Science, Beihai, China

**Keywords:** *Manihot esculenta* Crantz, single-nucleotide polymorphism (SNP), genetic diversity, population structure, genome-wide association analysis (GWAS)

## Abstract

Cassava (*Manihot esculenta* Crantz) is a major tuberous crop produced worldwide. In this study, we sequenced 158 diverse cassava varieties and identified 349,827 single-nucleotide polymorphisms (SNPs) and indels. In each chromosome, the number of SNPs and the physical length of the respective chromosome were in agreement. Population structure analysis indicated that this panel can be divided into three subgroups. Genetic diversity analysis indicated that the average nucleotide diversity of the panel was 1.21 × 10^-4^ for all sampled landraces. This average nucleotide diversity was 1.97 × 10^-4^, 1.01 × 10^-4^, and 1.89 × 10^-4^ for subgroups 1, 2, and 3, respectively. Genome-wide linkage disequilibrium (LD) analysis demonstrated that the average LD was about ∼8 kb. We evaluated 158 cassava varieties under 11 different environments. Finally, we identified 36 loci that were related to 11 agronomic traits by genome-wide association analyses. Four loci were associated with two traits, and 62 candidate genes were identified in the peak SNP sites. We found that 40 of these genes showed different expression profiles in different tissues. Of the candidate genes related to storage roots, Manes.13G023300, Manes.16G000800, Manes.02G154700, Manes.02G192500, and Manes.09G099100 had higher expression levels in storage roots than in leaf and stem; on the other hand, of the candidate genes related to leaves, Manes.05G164500, Manes.05G164600, Manes.04G057300, Manes.01G202000, and Manes.03G186500 had higher expression levels in leaves than in storage roots and stem. This study provides basis for research on genetics and the genetic improvement of cassava.

## Introduction

Cassava (*Manihot esculenta* Crantz, 2*n* = 36) is one of the three major tuberous crops produced worldwide. Cassava, a crop introduced in China in the 1820s, is the main food consumed by approximately 600 million people in tropical and subtropical regions; this crop is also the main material for starch and alcohol production (Balagopalan et al., 2001). Cassava is highly heterozygous; its vegetative reproduction generates complex genetic background, which also results in high genetic variability. Cassava was selectively domesticated at 7,000–12,000 years ago (Allem, 1994); its selective domestication yields the following characteristics: high mass accumulation, high starch rate, and the ability to grow and yield in unfavorable conditions, such as poor soil fertility and low rainfall. However, the precise mechanism underlying these characteristics requires further study. Molecular marker-assisted breeding is a feasible way to explore several beneficial allele variations through the selection of appropriate materials and desired phenotypic traits to locate desired functional genes or regions.

Given the development in novel generation sequencing technology and the completion of cassava genome draft (Bredeson et al., 2016), resequencing of cassava segregation population succeeded. Single-nucleotide polymorphisms (SNPs) and indels are also extensively developed. Furthermore, genetic maps with high-density molecular markers have since been constructed. The first high-density genetic map with 6,756 SNPs markers using genotyping by sequencing (GBS) technique in F1 segregation population of 180 varieties was made (Rabbi et al., 2014). In previous study, researchers obtained many expressed sequence tag(EST) SNP sites through transcriptome sequencing of 16 cassava varieties; they also used a method targeting enrichment to perform sequencing and genotype analysis on the F1 segregation population and consequently obtained a genetic linkage map containing 2,110 EST-SNPs (Pootakham et al., 2014). A new technology named amplified-fragment SNP and methylation (AFSM) was developed, which can simultaneously distinguish polymorphisms, such as SNPs, indels, and methylation sites between samples; based on this technology, a genetic map containing 3,032 AFSM markers (including 2,331 SNPs, 537 indels, and 164 methylation sites) was constructed by sequencing 85 F1 segregation groups (Xia et al., 2014). The International Cassava Genetic Map Consortium constructed a high-density molecular marker linkage map containing 22,403 SNP markers using GBS technology and 10 cassava segregation populations (International Cassava Genetic Map Consortium [ICGMC], 2015). Furthermore, a new cassava haplotype map (HapMapII) was constructed, which contained 25.9 million SNPs and 1.9 million insertions/deletions, at the same time, population genetic analysis and deleterious mutations in asexual reproduction were studied in this paper (Ramu et al., 2017; Ramu et al., unpublished). These genetic maps with high-density molecular markers are utilized in QTL mapping of several crucial traits, such as starch-related (Pootakham et al., 2014; Sraphet et al., 2016), disease-resistant (Kulakow et al., 2013; Rabbi et al., 2014; Boonchanawiwat et al., 2016), cold resistance-related (Zou et al., 2017), drought-related (Wang et al., 2017), and yield-related traits (Balyejusa et al., 2007; Sraphet et al., 2016). All these genetic maps are based on parent genetic groups, and they exhibit disadvantages, such as low resolution and limited availability. To date, only few reports regarding the QTL mapping of key genes in cassava have been published.

Genome-wide association analysis (GWAS) locates all the quantitative trait loci affecting phenotype using a sufficient amount of markers and linkage disequilibrium (LD) between alleles (Nordborg and Weigel, 2008). With the development in high-throughput technology and the reduced sequencing cost, GWAS has become a potential method for genetic dissection of complex traits. Nowadays, GWAS has been used in many plants, such as rice (Huang et al., 2010, 2011; Zhao et al., 2011), maize (Kump et al., 2011; Li et al., 2013), *Arabidopsis thaliana* (Atwell et al., 2010), and millet (Jia et al., 2013); consequently, the location of a number of excellent alleles has been determined. Appropriate genetic resources, including chromosome-scale reference genome and integrated linkage map, have been identified in cassava. Hence, GWAS has been utilized in different cassava populations; related articles on several phenotypes, particularly dry weight content (Rabbi et al., 2017), carotenoid content (Rabbi et al., 2017), and disease-resistant traits (Kulakow et al., 2013; Wolfe et al., 2016), have also been published.

In the present study, a total of 158 germplasms were collected from the Chinese Cassava Germplasm Resources; these germplasms were genotyped using of AFSM technology (Xia et al., 2014). On the basis of phenotypic identification of 11 agronomic traits at 3 years and four locations, GWAS was performed to identify association loci and candidate genes, and the tissue-specific expression patterns of these candidate genes were analyzed by RNA-seq.

## Materials and Methods

### Sample Collection

**FIGURE 1:**
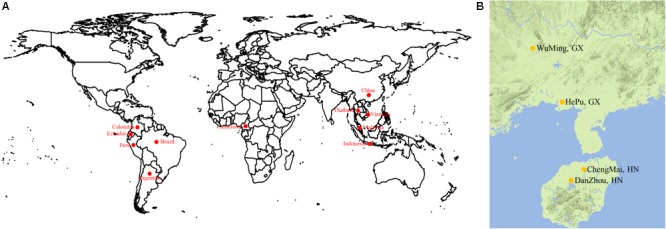
**(A)** Geographical distribution of varieties. **(B)** Cassava accessions were grown with three replications in four experimental farms.

A total of 158 cassava accessions were collected from ITBB CATAS and utilized for association analysis. Among them, 58 accessions originated from South America (including Argentina, Brazil, Ecuador, Colombia, and Peru), 24 from Southeast Asia (including Thailand, Vietnam, Indonesia, and Malaysia), 61 from China, 4 from Africa, and 11 from unknown regions (unknown; **Figure 1A** and **Supplementary Table S1**).

### Experimental Design and Trait Measurement

A randomized complete block design was used to grow the 158 accessions. Each accession was grown with three replications in an experimental farm at Hepu, Guangxi Province (108.51°E, 21.27°N); Wuming, Guangxi Province (107.49°E, 22.59°N); Danzhou, Hainan Province (109.5E, 19.5N); and Chengmai, Hainan Province (110.00°E, 19.75°N) in the 2013/2014/2015 growing seasons (**Figure 1B**). Each accession was grown in a plot with one row, and each row consisted of five or six plants. The distance between plants was 0.8 m. A total of 11 agronomic traits were finally selected. Of which, lobular length is the length of the maximum length of the intermediate lobe; the width of the lobular is the widest part of the middle lobe; the petiole length is the length of the petiole; the Leaf aspect ratio is the ratio of lobular length to the width of the leaflet; the first branch height is the height from ground to the first branch of the main stem; Stem diameter is the diameter of the main stem measuring 5 cm from the ground; the number of the storage roots refers to all the roots of a single plant; the storage roots weight means the weight of all the roots of a single plant; the dry matter content means the ratio of the weight of sliced and dried 200 g of fresh root to the weight of fresh root; dry matter weight means the product of fresh root and dry mater content; the starch content was determined by the method of optical rotation.

Given that the association panel traits were studied in multiple environments with three replications, a R script^1^ was utilized to achieve the best linear unbiased prediction (BLUP) of each trait in each line. This script is based on a linear model described by previous study (Merk et al., 2012). The obtained values were used as phenotypes for association analysis.

### DNA Preparation and Sequencing

Libraries were constructed through the CTAB method using DNA prepared from the fresh leaves of the 158 cassava varieties (Murray and Thompson, 1980). AFSM technology was conducted according to the method of previous study (Xia et al., 2014). All libraries underwent high-throughput sequencing in an Illumina HiSeq2000 apparatus (Illumina Inc., San Diego, CA, United States).

### SNP Calling and Annotation

Custom Perl scripts^2^ were used to process raw Illumina sequence reads. Processing was performed to optimize read numbers and reduce artifacts within data. Barcodes were used to assign the obtained sequences to individual samples; these sequences were also reduced to 2 × 132 bp. Distinct tags present in more than five different lines were determined in the complete set of reads. Bowtie 2 (Langmead and Salzberg, 2012) was utilized to align the filtered sequence reads to the AM560 cassava reference genome, which was obtained from JGI. SAMtools (Li et al., 2009) and VCFtools^3^ were used to identify SNPs and indels.

The SNPs and indels were annotated on the basis of the AM560 genome (obtained from JGI) through snpEff (Pablo et al., 2012). SNPs were categorized as being in intergenic regions, untranslated region (3′ and 5′ UTR), intronic, and coding sequences (CDS). Furthermore, SNPs in CDS were classified as synonymous or nonsynonymous. Indels in exons were categorized according to their frameshift effect.

### Population Genetics Analysis

Neighbor-joining method and a distance matrix calculated in PHYLIP^4^ were used to construct a phylogenetic tree, which is displayed in FigTree^5^. The genetic relatedness between individuals was assessed by using all the SNPs; assessment was carried out using principal component analysis (PCA) with GCTA tool (Yang et al., 2011). We calculated the eigenvector decomposition of the matrix in R using eigenfunction, and the PCA was plotted using these values. Population structure analysis was conducted using ADMIXTURE (Alexander et al., 2009). Analysis was performed by adopting the maximum likelihood method. Prior to using ADMIXTURE, we used PLINK 1.9 beta (Shaun et al., 2007) to obtain the required data files. Input parameter *K* varied from 1 to 12, which represented the assumed groups of simulated population in ancient times.

The VCFtools^3^ was used to calculate the genetic diversity (π) and population pairwise *F*-statistics (*F*_ST_). According to the standard description (Dunia et al., 2011), differentiation does not exist between subpopulations when *F*_ST_ = 0; however, subpopulations are completely differentiated when *F*_ST_ = 1. When *F*_ST_ < 0.05, populations are considered with little differentiation. Moderate, strong, and very strong differentiations occur when 0.05 < *F*_ST_ < 0.15, 0.15 < *F*_ST_ < 0.25, and *F*_ST_ > 0.25, respectively.

In the total panel and for each subgroup (as determined by the population structure), *r*^2^-values were used to determine the genome-wide LD by pairwise comparisons among the 25,989 SNP markers. The *r*^2^-value was obtained for all SNP pairs using the Plink 1.9 beta software (Shaun et al., 2007).

### GWAS

In this study, we used 25,989 high-quality SNPs and indels (MAF > 0.05 and HWE > 0.001) (Pongpanich et al., 2010) to perform GWAS on traits in 158 accessions. Association analyses were performed with TASSEL 5.0 (Bradbury et al., 2007) using compressed mixed linear model (MLM; P + G + Q + K) and simple model. Kinship was observed in all these SNPs. The significant association threshold was 1/*n*, where *n* is the total number of SNPs and indels. SAMtools was used for manual verification of regions with significant association from the aligned resequencing reads against the AM560 genome (obtained from JGI).

### Gene Annotation and Its Expression Patterns in Different Tissues

Based on the SNP annotation and LD decay of cassava, we take the gene of the association site itself as the candidate gene; if this site is located at the upstream and downstream of other genes, the upstream and downstream genes are also taken as candidate genes; if this site is located in the interval, the nearest upstream and downstream genes of the site are selected as candidate genes. The gene codes in the cassava genome were used to search the transcriptomics obtained from a gene bank; these gene codes were fresh leaf (FL), stem, early storage root (ETR), medium storage root (MTR), and later storage root (LTR) data with the following genotypes: KU50, Arg7, and W14. The RNA-seq data are available in a previous article (Wang et al., 2014). The NCBI login numbers include: SRX845399, SRX849491, SRX849498, SRX850754, SRX850774, SRX850863, SRX850861, SRX850870, and SRX850901. Log_10_-based FPKM-changed fold values were used to draw a heatmap in R using pheatmap package^6^.

## Results

### Genotyping of 158 Cassava Accessions

**FIGURE 2:**
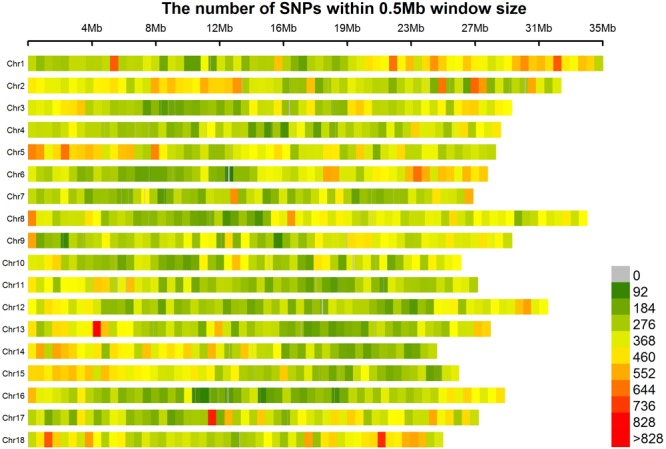
SNP distributions on 18 chromosomes of cassava. Horizontal axis displays the chromosome length; 0–828 legend insert indicates the SNP density.

We generated 120 Gbp of sequence from the 158 cassava accessions. When the sequencing reads from each sample were mapped to the reference genome of AM560 (obtained from JGI), more than 0.6 billion of 150 bp paired-end reads was generated (**Supplementary Table S2**). The mapping results of the 158 accessions were utilized to identify the SNPs of each accession using SAMtools (Li et al., 2009). A total of 349,827 SNP sand indels (including 293,008 SNPs and 56,819 indels) were detected. All the 349,827 SNPs covered all the 18 chromosomes. Chromosomes 1 (25,592 SNPs) and 2 (24,445 SNPs) presented the largest number of SNPs in sequence; the smallest number of SNPs was observed in chromosome 7 (16,275 SNPs). The number of SNPs on each chromosome and the physical length of the respective chromosome were in agreement. Approximately 1.48 kb/SNP was the average marker density on the whole genome. Chromosome 16 showed the lowest SNP marker density (1.76 kb/SNP), whereas chromosome 1 and 8 presented the highest marker density (1.27 kb/SNP) (**Figure 2** and **Supplementary Table S3**).

All SNPs and indels were filtered using cut-off values of MAF > 0.05 and HWE > 0.001 (Pongpanich et al., 2010); finally, 25,989 SNPs and indels (including 19,850 SNPs and 6,139 indels) were selected. The SNP diversity in the entire collection is expressed by a PIC value, and results are summarized in **Supplementary Table S4**. The average PIC value of the whole genome was approximately 0.162. About 90% of the SNPs in each chromosome, except for chromosomes 4, 7, and 17, displayed PIC values of more than 0.1. Chromosomes 1 and 7 exhibited the highest (nearly 0.168) and lowest (only 0.155) mean PIC values, respectively.

Among the identified SNPs, 7,420 (37.4%) were found in the gene region, and 4,421 SNPs (22.2%) were observed in CDS (**Table 1**). A total of 2,183 SNPs in the CDS were synonymous, and 2,238 were nonsynonymous. Consequently, the amino acid was altered. Thus, in the genome, the ratio of the number of nonsynonymous to synonymous SNPs was 1.03, which is higher than that of *Arabidopsis* (0.83) (Clark et al., 2007) and lower than those of soybean (1.37) (Honming et al., 2010) and rice (1.29) (Xu et al., 2012). Among the identified indels, 3,524 (57.4%) were located in the gene regions, and 1,908 (31.8%) caused frame shifts.

**Table 1:** Summary of single-nucleotide polymorphisms (SNPs) and indels.

Total SNPs	Intergenic	Untranslated region (UTR)		Intronic	Coding sequences			Nonsyn/syn ratio
		3′ UTR	5′ UTR		Total	Nonsynonymous	Synonymous	
19850	12430	684	974	1341	4421	2183	2238	1.03

**Total Indels**	**Intergenic**	**UTR**		**Intronic**	**Coding sequences**		
		**3′ UTR**	**5′ UTR**		**Total**	**Frameshift indel**	**Nonframeshift indel**	

6139	2615	277	415	460	2372	1908	464	

### Cassava Population Structure Analysis

**FIGURE 3:**
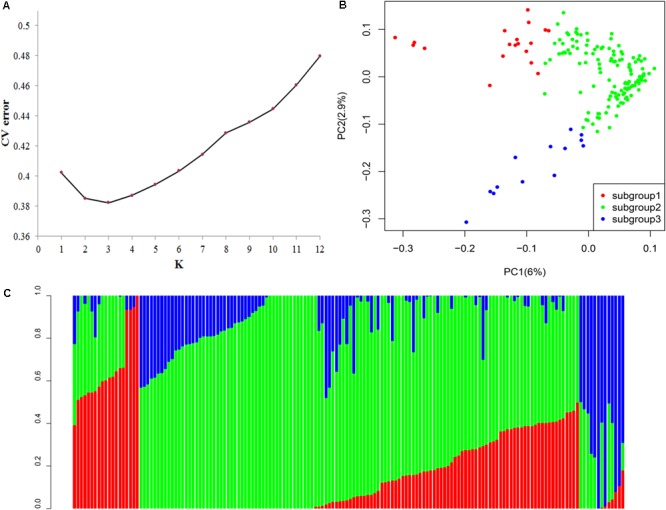
**(A)** Population structure analysis on 158 cassava accessions using ADMIXTURE. Estimated cross-validation error of possible clusters (*K*) from 1 to 12. **(B)** Population structure based on *K* = 3. In the panel, each individual is indicated with a vertical bar partitioned into three colored segments, and their respective lengths represent the proportion of the individual’s genome in a given group: red, green, and blue represent subgroups 1, 2, and 3, respectively. **(C)** Principal component analysis (PCA) of the population for 158 cassava accessions based on 25989 SNPs and indels. One dot represents each individual; the symbol color corresponds to the assigned subgroup classification. PCA plots of the same cassava germplasm collection according to subgroups, as identified by ADMIXTURE.

All SNPs were utilized to calculate the population structure of the association panel using ADMIXTURE. According to clustering inference with possible cluster range (*K*) of 1–12, the most significant change was observed when *K* = 3 (**Figure 3A**). These parameters indicated that the 158 genotypes can be classified into three groups. We used a membership threshold probability with maximum value. Additionally, 19, 126, and 13 lines were assigned into subgroups 1, 2, and 3, respectively (**Figure 3B**). Most subgroup 1 accessions came from South America (10), and nine accessions came from China (4), Southeast Asia (4), and Africa (1). Subgroup 2 accessions were mainly collected from China (51) and South America (41), and others came from Africa (3), Southeast Asia (20), and unknown regions (11). Subgroup 3 accessions were collected from China (6) and South America (7).

The PCA based on all the genome-wide SNPs demonstrated that 6 and 2.9% of the genetic variance was ascribed to the first two principal components, respectively. The three subgroups in the cassava panel along the PC1 axis were significantly correlated, as inferred by using ADMIXTURE (**Figure 3C**). The neighbor-joining phylogenetic tree was constructed to determine the genetic relationships among cassava accessions in the panel. No significant connection existed between the genetic relationships and geographical origins (**Supplementary Figure S1a**). This condition may be due to that the cassava promotion is relatively short, and no geographical differences exist.

### Genetic Diversity Revealed by SNP Markers

According to the SNP data, the genetic diversity (π) among all sampled landraces was 1.21 × 10^-4^, and 1.97 × 10^-4^, 1.01 × 10^-4^, and 1.89 × 10^-4^ values were obtained for subgroups 1, 2, and 3, respectively. Subgroup 2 showed less genetic diversity than those of subgroups 1 and 3, thereby indicating a loss in genetic diversity. The population differentiation statistic (*F*_ST_) between the three subgroups presented different population differentiation levels. The corresponding *F*_ST_ values between the subgroups ranged from moderate for subgroup 2 versus subgroup 1 (0.07) and subgroup 2 versus subgroup 3 (0.063) to little (0.034) for subgroup 1 versus subgroup 3 (**Supplementary Figure S1b**).

The highly heterozygous genotypes (heterozygosity = 0.14) agreed well with the asexual propagation of cassava. Subgroup 2 showed lower average heterozygosity (0.11) than those in subgroups 1 (0.26) and 3 (0.25). The genetic distance among the 158 cassava accessions was in the range of 0.03–0.32, with an average of 0.15. Nevertheless, subgroup 2 showed the lowest average genetic distance (0.13) and the smallest range (0.03–0.23). By contrast, subgroups 1 and 3 presented similar average genetic distance (0.24 and 0.22, respectively), which ranged from 0.12 to 0.31 and from 0.11 to 0.28, respectively.

### LD Across Whole Cassava Genome

The LD (indicated by *r*^2^) decreased with physical distance between SNPs in all cassava groups (**Supplementary Figure S2**). The LD level measured in each group is the chromosomal distance when LD decreased to 0.2. In agreement with results for other crops, the LD level in cassava in subgroup 2 was lower (6 kb) than those in subgroups 1 (8.5 kb) and 3 (9 kb). The observed LD level in cultivated cassava groups was 8 kb, which was lower than that in cultivated maize (30 kb) (Hufford et al., 2012), cultivated rice (123 kb in *Oryza indica*) (Huang et al., 2010), or cultivated soybean (133 kb) (Zhou et al., 2015), but higher than that of inbred maize (1.5 kb) (Remington et al., 2001) and potato (1 kb) (Simko, 2004).

### Phenotypic Variations of Measured Quantitative Traits

All the 11 traits in the cassava association panel, which comprised 158 accessions, were evaluated with three replications in four locations for 3 years. We detected extensive phenotypic variations, as displayed in the descriptive statistics in **Supplementary Table S5**. The leaf aspect ratio, which varied from 3.01 to 11.55 (average, 4.95), showed the minimum coefficient of variation of 30% in Hepu (2015); in Chengmai (2015), the leaf aspect ratio, which varied from 2.87 to 14.67 (average, 4.95), displayed the maximum coefficient of variation of 40%; the average variation coefficient was 4.65 (35%), 4.18 (38%), 4.23 (35%), and 4.44 (31%) in Hepu (2013), Chengmai (2014), and Wuming (2014, 2015), respectively. This trait was observed stable in different environments. Dry mass weight varied from 0.15 to 0.99 (average, 0.5), and its minimum variation coefficient was 34% in Chengmai (2013). In Wuming (2013), the dry mass weight varied from 0.12 to 2.69 (average, 0.88) and showed the maximum variation coefficient of 53%; the average variation coefficient was 0.78 (47%), 1.09 (39%), 0.35 (54%), 1.26 (42%), 3.12 (42%), 0.61 (35%), 0.76 (52%), 1.26 (45%), and 1.15 (49%) in Hepu (2013, 2015), Chengmai (2013, 2014, 2015), Danzhou (2014, 2015), and Wuming (2014, 2015), respectively. Dry mass weight was highly diverse in different environments.

**FIGURE 4:**
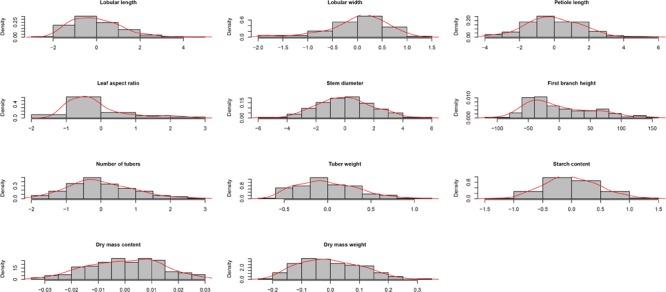
Density distribution of variation in 11 traits (best linear unbiased prediction values) in cassava germplasm.

We also calculated the broad-sense heritability of each trait to determine the relationship between genetic and environmental factors during trait variation. Among these 11 traits, leaf length and width ratio, leaflet width, and first branch height showed the largest broad-sense heritability of 0.92, 0.86, and 0.79, respectively. The phenotype variation values of these traits were low in different years and locations, which indicated that genetic factors were the main reasons for these traits rather than environmental factors. Dry mass weight, storage roots weight, and petiole length presented the smallest broad-sense heritability of 0.38, 0.49, and 0.54, respectively; the phenotype variation values of these traits varied among different years and locations, which suggested that these traits were influenced with environmental factors. The phenotype variation values of other traits ranged between 0.47 and 0.73, and these traits varied under different environments. These results demonstrated that environmental factors affected the phenotypes of cassava. Thus, we calculated the BLUP value of each phenotype as the phenotype value for GWAS analysis to reduce the effect of environmental factors on phenotype variation (**Figure 4**).

### GWAS on 11 Important Traits

**FIGURE 5:**
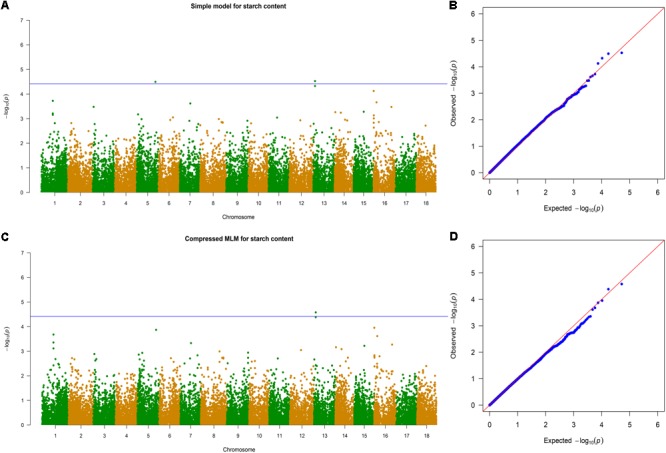
Genome-wide association analysis of starch content. **(A)** Manhattan plots of simple model for starch content. Negative log_10_-transformed P values from a genome-wide scan are plotted against position on each of the 18 chromosomes. Genome-wide significance threshold is depicted as a blue horizontal dashed line. **(B)** Quantile–quantile plot of the simple model for starch content. **(C)** Manhattan plots of compressed MLM for traits as in **(A)**. **(D)** Quantile–quantile plot of the compressed MLM for starch content.

The 11 traits were classified into four categories, namely, leaf characteristics (lobular length, lobular width, petiole length, and leaf aspect ratio), morphological characteristics (stem diameter and first branch height), yield components (number of storage roots, storage roots weight, and dry mass weight), and storage root quality (dry matter content and starch content). These traits were considered for GWAS with whole-genome diverse SNP markers. Association signals were identified using the simple model and the compressed MLM. The compressed MLM approach significantly decreased the false positives, as shown in quantile–quantile plots (**Figure 5** and **Supplementary Figures S3–S12**). This approach considered the genome-wide patterns of genetic relatedness. A total of 17 and 34 association signals were identified with *P* < 3.85 × 10^-5^ using the compressed MLM (**Table 2**) and simple model (**Supplementary Table S6**). A total of 36 associations were detected for the 11 agronomic traits. **Figure 5** and **Supplementary Figures S3–S12** show the Manhattan plots for both models of all the traits. Detailed information about all significant associations is summarized in **Supplementary Table S7**. Storage root quality showed four association signals. Approximately 12% of phenotypic variance on average was attributed to the peak SNPs at identified loci. The seven peak SNPs, which were identified from yield component, explained ∼14.95% of phenotypic variance on average (12.9–15.28% for different loci). Morphological characteristics exhibited 11 association signals and explained 12.23–20.86% of the phenotypic variance. A total of 14 association signals were identified from leaf characteristics, which explained 11.86–22.56% of the phenotypic variance with an average 16%.

**Table 2:** Genome-wide significant association signals of 11 agronomic traits from the compressed mixed linear model (MLM).

Trait	Chr.	Position	Major allele	Minor allele	MAF	-log_10_ P (MLM model)	*r*^2^
Starch content	13	2226552	G	A	0.12	4.58	0.12
Dry mass content	14	785145	A	C	0.08	4.6	0.14
Dry mass weight	2	15786746	G	A	0.07	4.46	0.13
Storage roots weight	2	12880992	A	G	0.19	4.56	0.13
Number of storage roots	5	14994529	T	A	0.08	4.85	0.15
Number of storage roots	9	21856881	A	G	0.09	4.99	0.15
Lobular width	5	22904128	A	T	0.14	4.44	0.16
Lobular width	3	25192695	T	C	0.05	5.25	0.19
Lobular width	10	2651408	A	C	0.15	4.82	0.17
Leaf aspect ratio	3	25192695	T	C	0.05	6.76	0.23
Leaf aspect ratio	6	9070938	A	G	0.1	5.52	0.19
Leaf aspect ratio	14	4417235	GT	G	0.09	5.18	0.17
Leaf aspect ratio	1	28193343	T	C	0.08	4.81	0.16
Leaf aspect ratio	3	27223440	C	G	0.14	4.73	0.16
Leaf aspect ratio	10	2651408	A	C	0.15	4.68	0.16
Stem diameter	3	5868851	G	A	0.1	4.82	0.19
Stem diameter	7	24605269	T	A	0.14	4.86	0.19

*Chr. Chromosome; NA, Not available; MAF, Minor allele freq.*

Gene annotation information was used to determine the putative function of genes around associated loci (**Supplementary Table S7**). A total of 62 candidate genes were identified in the peak SNP sites (or adjacent to these sites) of 36 associated loci (**Table 3**). Six candidate genes were related to storage root quality; these genes included Manes.13G023300 (beta-1,4-N-acetylglucosaminyl transferase family protein), which participates in polysaccharide synthesis; Manes.16G000700 (AAA-type ATPase family protein), which is a generator of mechanical force and a key player in protein degradation throughout evolution (Bar-Nun and Glickman, 2012); Manes.16G000800 (leucine-rich repeat family protein) for growth regulation and development, immunity, signal transduction, and stress responses of plants (Sun et al., 2017); Manes.13G023400 [NAD(P)-linked oxidoreductase superfamily protein]; Manes.05G177800 (domain with unknown function); and Manes.14G007500 (glyceraldehyde-3-phosphate dehydrogenase of plastid 2), which is related to glycolysis.

**Table 3:** Associated loci and candidate genes according to gene annotation.

Trait	Chr.	Position	Candidate genes	Description
Starch content	13	2226552	Manes.13G023300, Manes.13G023400	Beta-1,4-N-acetylglucosaminyl transferase family protein, NAD(P)-linked oxidoreductase superfamily protein
	5	24421682	Manes.05G177800	Domain of unknown function (DUF303)
Dry mass content	14	785145	Manes.14G007500	Glyceraldehyde-3-phosphate dehydrogenase of plastid 2(GAPCP-2)
	16	96080	Manes.16G000700, Manes.16G000800	AAA-type ATPase family protein, leucine-rich repeat (LRR) family protein
Dry mass weight	2	15786746	Manes.02G192500, Manes.02G192600	Expressed protein, protease-associated RING/U-box zinc finger family protein
	2	12880992	Manes.02G169700, Manes.02G169800	Nodulin MtN3 family protein, expressed protein
	2	11597069	Manes.02G154700, Manes.02G154800	Glycosyl hydrolase family protein, calcineurin-like metallo-phosphoesterase superfamily protein
Number of storage roots	5	14994529	Manes.05G125100, Manes.05G125200, Manes.05G125300	Inositol requiring 1-1, glyceraldehyde-3-phosphate dehydrogenase B subunit, expressed protein
	4	13096211	Manes.04G057900, Manes.04G058000	Expressed protein, expressed protein
	9	21856881	Manes.09G099100, Manes.09G099200, Manes.09G099300	Expressed protein, Ctr copper transporter family, HSP20-like chaperones superfamily protein
Storage roots weight	2	12880992	Manes.02G169700, Manes.02G169800	Nodulin MtN3 family protein, expressed protein
Stem diameter	3	5868851	Manes.03G059800	Expressed protein
	3	26144665	Manes.03G170000, Manes.03G169900	Protein of unknown function (DUF2361), glycosyl hydrolase family 47 protein
	14	1904613	Manes.14G021000, Manes.14G020900	NADH-ubiquinone oxidoreductase-related, SNARE-like superfamily protein
	7	24605269	Manes.07G117800, Manes.07G117700	ROTUNDIFOLIA like 8, expressed protein
	7	915753	Manes.07G007700, Manes.07G007800	Reduced lateral root formation, sterol methyltransferase 1
	6	13802363	Manes.06G047300	Small nuclear RNA-activating complex (SNAPc), subunit SNAP43 protein
First branch height	9	5736110	Manes.09G041800, Manes.09G041900	Calcium-dependent protein kinase 20, ABC-2 type transporter family protein
	2	23221247	Manes.02G212100, Manes.02G212200	Pectin lyase-like superfamily protein, expressed protein
	3	6235785	Manes.03G061100, Manes.03G061200	Aspartate aminotransferase 3, tetraspanin family protein
	10	23491074	Manes.10G123100	Transmembrane nine 1
	9	26163242	Manes.09G143900, Manes.09G144000, Manes.09G143800	Expressed protein, expressed protein, *Arabidopsis* NAC domain containing protein 87
Lobular width	5	22904128	Manes.05G164600, Manes.05G164500	Ribosomal protein S9, MATE efflux family protein
	3	25192695	Manes.03G156800, Manes.03G156900, Manes.03G157000	Mitochondrial editing factor 22, Myb domain protein 305, AWPM-19-like family protein
	10	2651408	Manes.10G031200	OBF-binding protein 3
Lobular length	5	1945050	Manes.05G026500, Manes.05G026600, Manes.05G026700	Galactose mutarotase-like superfamily protein, expressed protein, G-box binding factor 3
	6	9070958	Manes.06G034000	26S proteasome regulatory subunit, putative
	18	1466764	Manes.18G018500	Histone superfamily protein
	4	12749959	Manes.04G057300	Aldehyde oxidase 2
petiole length	1	29478891	Manes.01G201900, Manes.01G202000	Basic helix-loop-helix DNA-binding superfamily protein, expressed protein
Leaf aspect ratio	3	25192695	Manes.03G156800, Manes.03G156900, Manes.03G157000	Mitochondrial editing factor 22, Myb domain protein 305, AWPM-19-like family protein
	6	9070938	Manes.06G034000	26S proteasome regulatory subunit, putative
	14	4417235	Manes.14G056200, Manes.14G056100, Manes.14G056300	MA3 domain-containing protein, MA3 domain-containing protein, expressed protein
	1	28193343	Manes.01G182700, Manes.01G182800, Manes.01G182900	Plant invertase/pectin methylesterase inhibitor superfamily, expressed protein, glycine-rich protein
	3	27223440	Manes.03G186400, Manes.03G186500	P-loop -containing nucleoside triphosphate hydrolase superfamily protein, alpha/beta-hydrolase superfamily protein
	10	2651408	Manes.10G031200	OBF-binding protein 3

**FIGURE 6:**
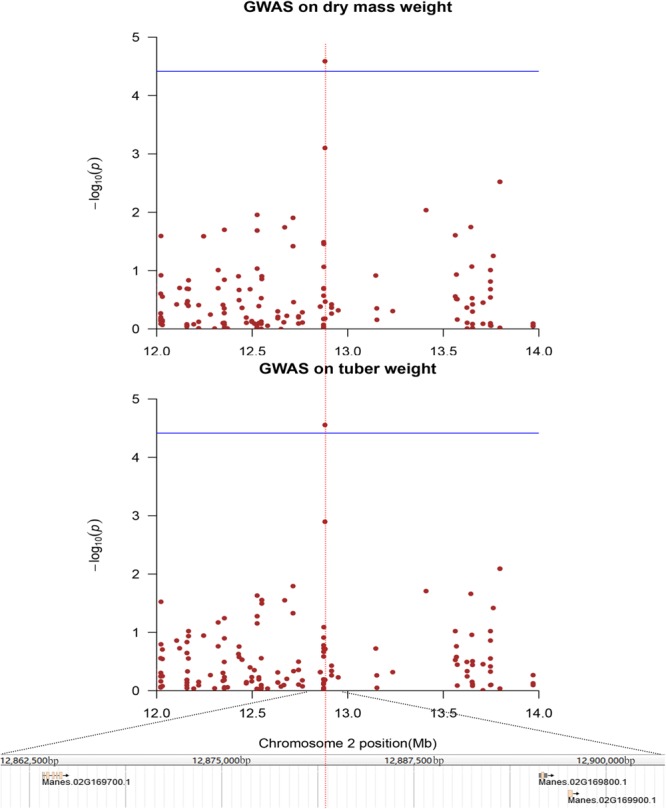
Genome-wide association analysis of dry mass weight and storage roots weight in 158 cassava accessions using the SNPs detected on chromosome 2. Genomic position (*X*-axis) is plotted against its significance expressed as –log_10_ P value (*Y*-axis). Genomic position covers 1 Mb on either side of the peak SNP, as shown in a black dashed vertical line. Genome-wide significance threshold is depicted as a blue horizontal dashed line. Annotated candidate genes are indicated in pink boxes below the graph.

Among the yield component-related candidate genes, one SNP locus, which was located in chromosome 2, was associated with fresh weight and dry mass weight (**Figure 6**). Two candidate genes, including an unknown functional protein (Manes.02G169800, expressed protein) and a functional protein (Manes.02G169700, nodulin MtN3 family protein), were annotated; a transmembrane receptor protein, which may be related to the cell wall formation and maintenance of cell integrity, is also obtained by annotations (Guan et al., 2008; Quiapim et al., 2009). Two SNP loci were mapped to lobular width and leaf aspect ratios. Four genes were obtained by annotation; these genes included Manes.03G156800 (mitochondrial editing factor 22); Manes.03G156900 (Myb domain protein 305), which is a Myb transcription factor crucial in regulating the development of secondary cellular walls and fiber biosynthesis (Liu et al., 2017); Manes.03G157000 (AWPM-19-like family protein), which encodes proteins that can be linked to the cellular membrane and transport function (Schatzberg et al., 2005; Wu et al., 2010); and Manes.10G031200 (OBF-binding protein 3), which is a Dof transcription factor and a SA-responsive gene (Kang et al., 2003) (**Supplementary Figures S13, S14**). One SNP locus was mapped to lobular length and leaf aspect ratios, and one gene called Manes.06G034000 (26S proteasome regulatory subunit, putative) was obtained through annotation (**Supplementary Figure S15**).

### Expression Pattern Analysis

**FIGURE 7:**
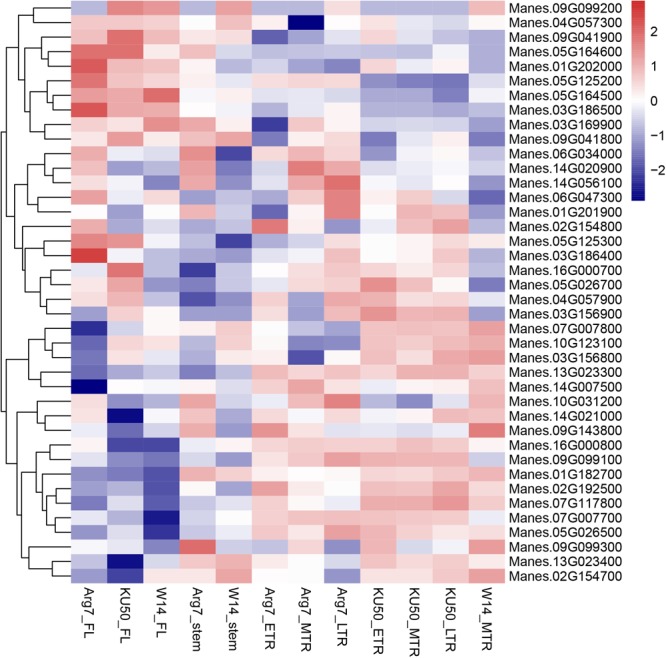
Expression profiles of associated genes in cassava. A heatmap was drawn using log_10_-based FPKM-changed fold values. The differential expression thresholds of significant up- and downregulation were 3.0 and –3.0, respectively.

Among the 62 candidate genes, 40 genes presented expression profiles in different cassava tissues according to previous RNA-seq data. We used log_10_-based FPKM-changed fold values to draw a heatmap (**Figure 7**), with 3.0 and -3.0 as the upper and lower limits, respectively. Among the candidate genes identified by yield components and storage root quality, the following genes showed high expression levels in storage root tissues: Manes.13G023300, Manes.16G000800, Manes.02G154700, Manes.02G192500, and Manes.09G099100. The expression levels of 11 genes related to morphological characteristics showed no variation in different tissues. The following genes, which are associated with leaf characteristics, exhibited high expression levels in leaves: Manes.05G164500, Manes.05G164600, Manes.04G057300, Manes.01G202000, and Manes.03G186500. The new genes identified in the present study are promising candidates for follow-up studies on the genetic architecture of these traits.

## Discussion

The GWAS on cassava landraces can be applied in simultaneous genetic mapping of multiple traits at a fine resolution. AFSM resequencing of cassava landraces obtains a large number of sequence polymorphisms and high association resolution in GWAS, despite the low rates of LD decay in cassava. Notably, simultaneous identification of polymorphisms, such as SNPs, indels, and methylation sites between samples, can be performed only through the AFSM technology. The method is successfully applied in other species, such as *M. esculenta* Crantz (Xia et al., 2014; Zou et al., 2017) and *Jatropha carcas* L. (Xia et al., 2018).

Hence, detailed knowledge about population structure in an association panel is crucial to prevent any sham association (Flintgarcia et al., 2005). Different populations are typically used to assess structures in cassava. For instance, the cassava panel along the PC1 axis can be divided into three subgroups according to pedigree or geography (Bredeson et al., 2016; Ramu et al., 2017; Ramu et al., unpublished). Similarly, in our study, cross-validation error determined a *K*-value of 3. We used a membership threshold probability with maximum value, and the panel was categorized into subgroups 1, 2, and 3. Nevertheless, the PCA cannot determine considerable variance in the PC1 axis, which indicated that the cassava panel was consisted of numerous admixed lines and a low-layer population structure. Differentiation between subgroups was further confirmed by the *F*_ST_ value (0.03–0.07). Thus, this panel showed low genetic differentiation. This study obtained results similar to those of previous results; the geographic subpopulations of cultivated cassava show low *F*_ST_ values among themselves (0.01–0.05) (Ramu et al., 2017; Ramu et al., unpublished).

The LD decay over a known genetic distance is crucial in the determination of the number and density of a molecular marker for GWAS and selection strategies (Mather et al., 2007). Cassava, as a predominantly cross-pollinated or clonal propagation species, should present a lower LD level than that of self-pollinated crops. In the previous study, the LD decay was ∼3 kb, which is the point at which the *r*^2^ decreases to 0.1 (Ramu et al., 2017; Ramu et al., unpublished), and in this study, the LD decay was ∼8 kb, which is similar to that of grape (< 10 kb) (Myles et al., 2011).

Phenotypic identification of cassava grown in suitable environment is very difficult because they are sensitive to typoon and rains; to reduce phenotypic error, we evaluated all the traits of the cassava panel in four locations for three years; the BLUP, which can be integrated on numerous environmental data, was used; the environmental effect was removed, and a stable individual genetic phenotype was obtained (Merk et al., 2012). Many candidate genes can be determined through GWAS, but most of them are new genes without functional verification. Therefore, gene expression pattern can show whether the gene possesses biological function. The gene expression results in the present study were obtained from previous independent research experiments (Wang et al., 2014). The expression profiles of specific tissues in different accessions can be used to identify candidate genes accurately through GWAS; for example, numerous expression quantitative trait loci are identified in maize by using kernel (Fu et al., 2013). These resources can also identify expressed quantitative trait loci at high resolution to further understand the gene regulatory network of the corresponding traits.

In this study, the GWAS potential is limited with the small population size and low genetic diversity from cassava samples. Cassava accessions for AFSM resequencing should be collected worldwide. Comprehensive phenotyping is currently in progress, and associations in this broad sampling can be studied in the future. Given that it allows the addition of new SNPs and the improvement of imputation efficiency at a low sequence coverage, genome sequencing is effective for GWAS in studies that aim to improve map resolution and new-allele identification through continuous population expansion. This study provides basis for a long-term collective exploration to determine valuable genes and alleles from the world germplasm collection and consequently improve cultivars.

## Author Contributions

WW and ZX conceived and designed the studies. SZ and ZX performed the data analysis. SZ wrote the manuscript. SZ, JY, CL, KL, MZ, SF, XC, JP, PM, CHL, XZ, ZL, CJL, QL, and ZX identified the phenotypes. WW and ZX revised the paper. All authors read and approved the final manuscript.

## Conflict of Interest Statement

The authors declare that the research was conducted in the absence of any commercial or financial relationships that could be construed as a potential conflict of interest.

## Footnotes

1 http://articles.extension.org/pages/61006

2 http://afsmseq.sourceforge.net/

3 http://vcftools.sourceforge.net/

4 http://evolution.genetics.washington.edu/phylip.html

5 http://tree.bio.ed.ac.uk/software/figtree/

6 https://CRAN.R-project.org/package=pheatmap

## Supplementary Material

FIGURE S1 Neighbor-joining phylogenetic tree of the 158 cassava accessions. Red, green, and blue indicate subgroups 1, 2, and 3, respectively. **(b)** Nucleotide diversity (π) and population divergence (*F*_ST_) across the three groups. Nucleotide diversity of the groups is represented by the value in each circle. Population divergence between two groups is indicated by the value on each line.

FIGURE S2 Genome-wide average linkage disequilibrium decay estimated from all cassava samples (black) and subgroups 1 (red), 2 (green), and 3 (blue).

FIGURE S3 Genome-wide association analyses on dry mass weight. **(a)** Manhattan plots of the simple model for dry mass weight. Negative log_10_-transformed P values from a genome-wide scan are plotted against position on each of the 18 chromosomes. Genome-wide significance threshold is depicted as a blue horizontal dashed line. **(b)** Quantile–quantile plot of the simple model for dry mass weight. **(c)** Manhattan plots of the compressed MLM for traits as in a. **(d)** Quantile–quantile plot of the compressed MLM for dry mass weight.

FIGURE S4 Genome-wide association analyses on dry mass content. **(a)** Manhattan plots of the simple model for dry mass content. Negative log_10_-transformed P values from a genome-wide scan are plotted against position on each of the 18 chromosomes. Genome-wide significance threshold is depicted as a blue horizontal dashed line. **(b)** Quantile–quantile plot of the simple model for dry mass content. **(c)** Manhattan plots of the compressed MLM for traits as in a. **(d)** Quantile–quantile plot of the compressed MLM for dry mass content.

FIGURE S5 Genome-wide association analyses on first branch height. **(a)** Manhattan plots of the simple model for first branch height. Negative log_10_-transformed P values from a genome-wide scan are plotted against position on each of the 18 chromosomes. Genome-wide significance threshold is depicted as a blue horizontal dashed line. **(b)** Quantile–quantile plot of the simple model for first branch height. **(c)** Manhattan plots of the compressed MLM for traits as in a. **(d)** Quantile–quantile plot of the compressed MLM for first branch height.

FIGURE S6 Genome-wide association analyses on leaf aspect ratio. **(a)** Manhattan plots of the simple model for leaf aspect ratio. Negative log_10_-transformed P values from a genome-wide scan are plotted against position on each of the 18 chromosomes. Genome-wide significance threshold is depicted as a blue horizontal dashed line. **(b)** Quantile–quantile plot of the simple model for leaf aspect ratio. **(c)** Manhattan plots of the compressed MLM for traits as in a. **(d)** Quantile–quantile plot of the compressed MLM for leaf aspect ratio.

FIGURE S7 Genome-wide association analyses on lobular length. **(a)** Manhattan plots of the simple model for lobular length. Negative log_10_-transformed P values from a genome-wide scan are plotted against position on each of the 18 chromosomes. Genome-wide significance threshold is depicted as a blue horizontal dashed line. **(b)** Quantile–quantile plot of the simple model for lobular length. **(c)** Manhattan plots of the compressed MLM for traits as in a. **(d)** Quantile–quantile plot of the compressed MLM for lobular length.

FIGURE S8 Genome-wide association analyses on lobular width. **(a)** Manhattan plots of the simple model for lobular width. Negative log_10_-transformed P values from a genome-wide scan are plotted against position on each of the 18 chromosomes. Genome-wide significance threshold is depicted as a blue horizontal dashed line. **(b)** Quantile–quantile plot of the simple model for lobular width. **(c)** Manhattan plots of the compressed MLM for lobular width as in a. **(d)** Quantile–quantile plot of the compressed MLM for lobular width.

FIGURE S9 Genome-wide association analyses on the number of storage roots. **(a)** Manhattan plots of the simple model for the number of storage roots. Negative log_10_-transformed P values from a genome-wide scan are plotted against position on each of the 18 chromosomes. Genome-wide significance threshold is depicted as a blue horizontal dashed line. **(b)** Quantile–quantile plot of the simple model for traits. **(c)** Manhattan plots of the compressed MLM for the number of storage roots as in a. **(d)** Quantile–quantile plot of the compressed MLM for the number of storage roots.

FIGURE S10 Genome-wide association analyses on petiole length. **(a)** Manhattan plots of the simple model for petiole length. Negative log_10_-transformed P values from a genome-wide scan are plotted against position on each of the 18 chromosomes. Genome-wide significance threshold is depicted as a blue horizontal dashed line. **(b)** Quantile–quantile plot of the simple model for traits. **(c)** Manhattan plots of the compressed MLM for petiole length as in a. **(d)** Quantile–quantile plot of the compressed MLM for petiole length.

FIGURE S11 Genome-wide association analyses on stem diameter. **(a)** Manhattan plots of the simple model for stem diameter. Negative log_10_-transformed P values from a genome-wide scan are plotted against position on each of the 18 chromosomes. Genome-wide significance threshold is depicted as a blue horizontal dashed line. **(b)** Quantile–quantile plot of the simple model for traits. **(c)** Manhattan plots of the compressed MLM for stem diameter as in a. **(d)** Quantile–quantile plot of the compressed MLM for stem diameter.

FIGURE S12 Genome-wide association analyses on storage roots weight. **(a)** Manhattan plots of the simple model for storage roots weight. Negative log_10_-transformed P values from a genome-wide scan are plotted against position on each of the 18 chromosomes. Genome-wide significance threshold is depicted as a blue horizontal dashed line. **(b)** Quantile–quantile plot of the simple model for storage roots weight. **(c)** Manhattan plots of the compressed MLM for traits as in a. **(d)** Quantile–quantile plot of the compressed MLM for storage roots weight.

FIGURE S13 Genome-wide association analysis on leaf aspect ratio and lobular width in 158 cassava accessions using the SNPs detected on Chromosome 3. Genomic position (*x* axis) is plotted against its significance expressed as -log_10_ P value (*y* axis). Genomic position covers 1 Mb on either side of the peak SNP, as shown in a black dashed vertical line. Genome-wide significance threshold is depicted as a blue horizontal dashed line. Annotated candidate genes are indicated in pink boxes below the graph.

FIGURE S14 Genome-wide association analysis on leaf aspect ratio and lobular width in 158 cassava accessions using the SNPs detected on Chromosome 10. Genomic position (*x* axis) is plotted against its significance expressed as -log_10_ P value (*y* axis). Genomic position covers 1 Mb on either side of the peak SNP, as shown in a black dashed vertical line. Genome-wide significance threshold is depicted as a blue horizontal dashed line. Annotated candidate genes are indicated in pink boxes below the graph.

FIGURE S15 Genome-wide association analysis on leaf aspect ratio and lobular length in 158 cassava accessions using the SNPs detected on Chromosome 6. Genomic position (*x* axis) is plotted against its significance expressed as -log_10_ P value (*y* axis). Genomic position covers 1 Mb on either side of the peak SNP, as shown in a black dashed vertical line. Genome-wide significance threshold is depicted in blue horizontal dashed line. Annotated candidate genes are indicated in pink boxes below the graph.

TABLE S1 List of cassava samples with the origin.

TABLE S2 Total reads in each accession.

TABLE S3 Summary of the number of SNPs mapped onto each chromosome.

TABLE S4 Summary of the PIC values in different chromosomes of the cassava.

TABLE S5 Phenotypic variations for 11 traits in this cassava panel.

TABLE S6 Genome-wide significant association signals of 11 agronomic traits from the simple model.

TABLE S7 Genome-wide significant association signals of 11 agronomic traits from the simple model and the compressed MLM.
