# Supplementary figures and images for: Genome-Wide Association Studies of 11 Agronomic Traits in Cassava (Manihot esculenta Crantz)
Source: Front Plant Sci. 2018 Apr 19;9:503. doi: 10.3389/fpls.2018.00503 (PMC5917017; doi:10.3389/fpls.2018.00503)

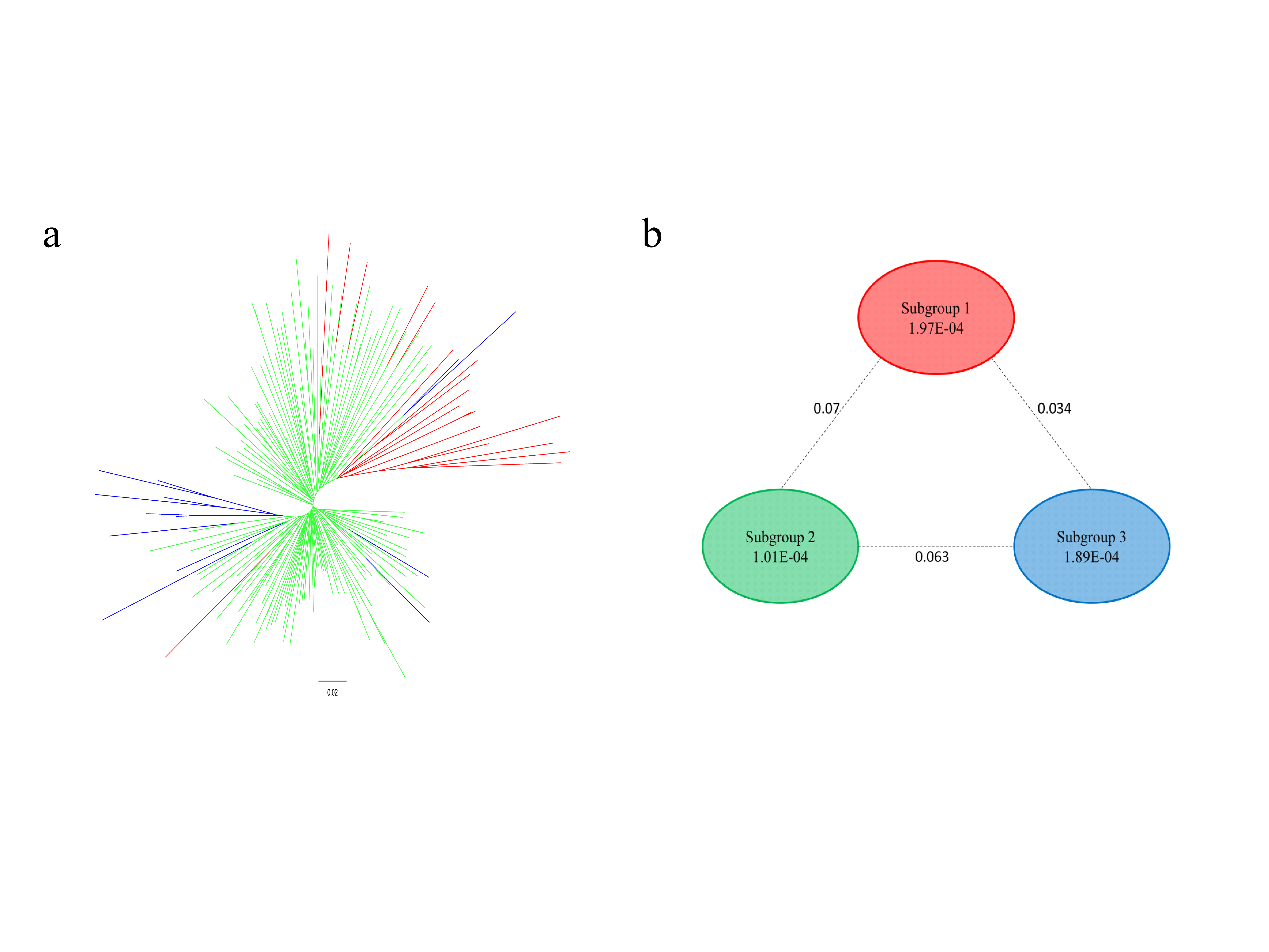

Supplement: FIGURE S1 — Neighbor-joining phylogenetic tree of the 158 cassava accessions. Red, green, and blue indicate subgroups 1, 2, and 3, respectively. (b) Nucleotide diversity (π) and population divergence (FST) across the three groups. Nucleotide diversity of the groups is represented by the value in each circle. Population divergence between two groups is indicated by the value on each line. [file Image_1.TIF]

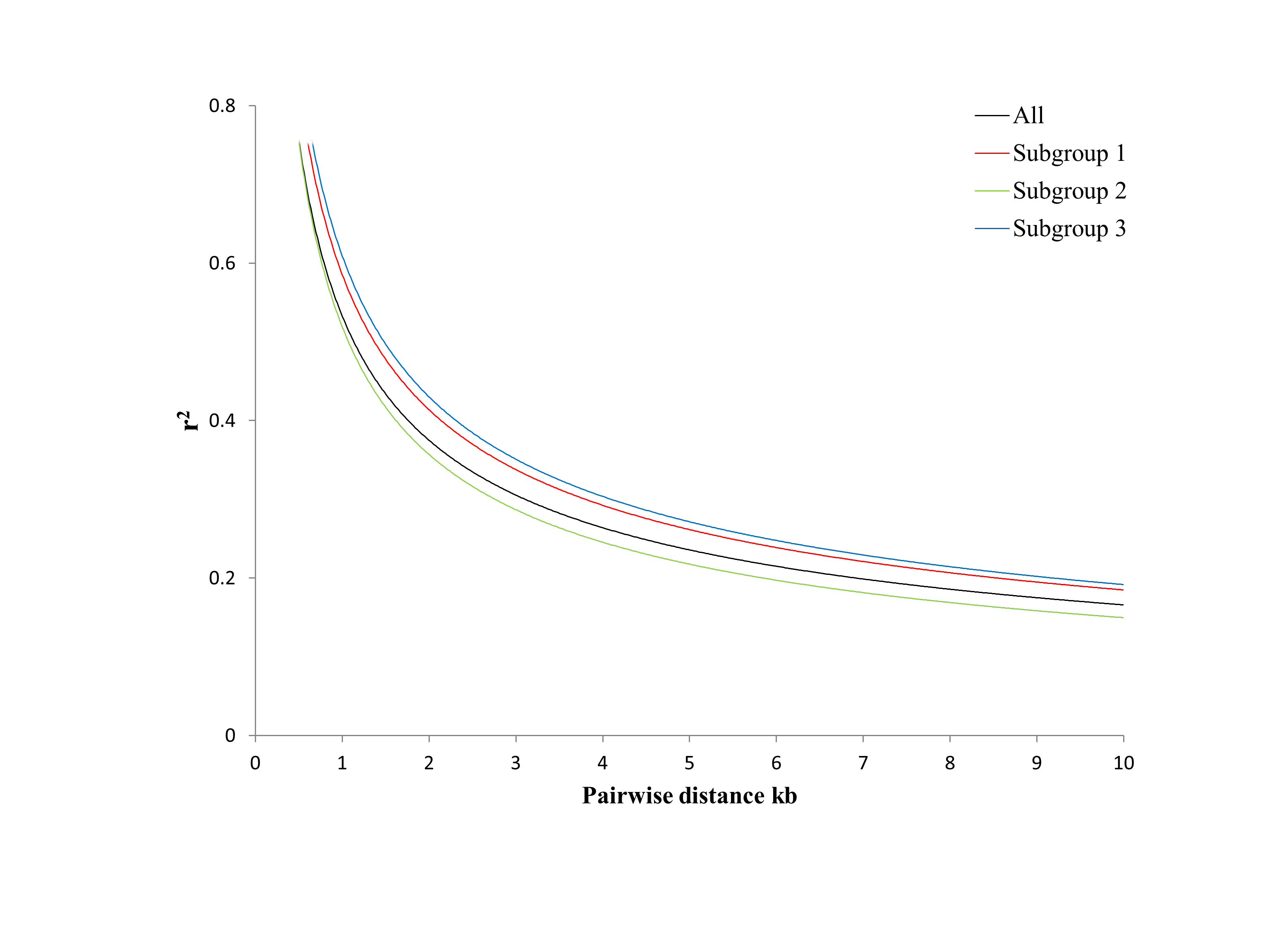

Supplement: FIGURE S2 — Genome-wide average linkage disequilibrium decay estimated from all cassava samples (black) and subgroups 1 (red), 2 (green), and 3 (blue). [file Image_2.TIF]

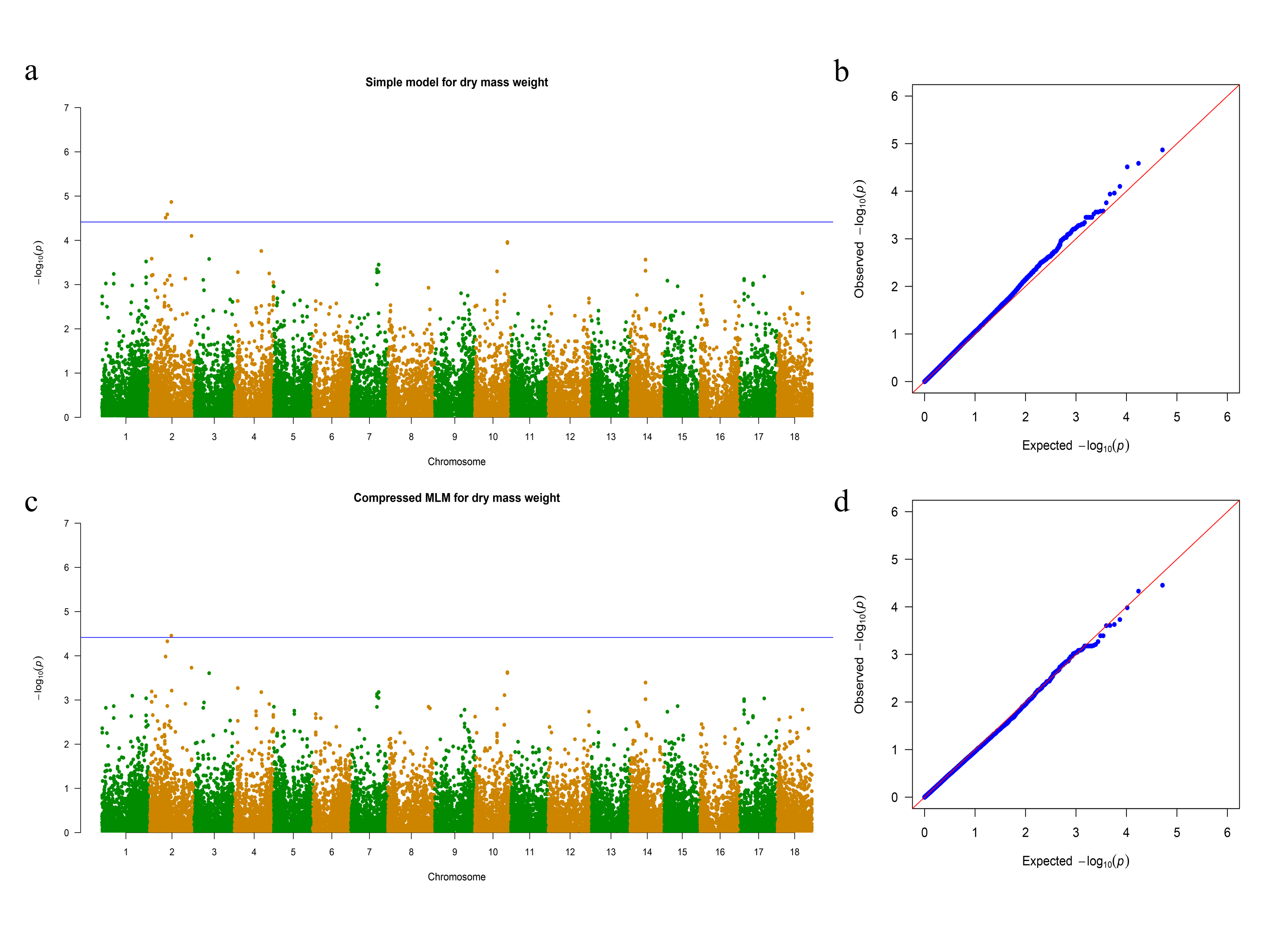

Supplement: FIGURE S3 — Genome-wide association analyses on dry mass weight. (a) Manhattan plots of the simple model for dry mass weight. Negative log10-transformed P values from a genome-wide scan are plotted against position on each of the 18 chromosomes. Genome-wide significance threshold is depicted as a blue horizontal dashed line. (b) Quantile–quantile plot of the simple model for dry mass weight. (c) Manhattan plots of the compressed MLM for traits as in a. (d) Quantile–quantile plot of the compressed MLM for dry mass weight. [file Image_3.TIF]

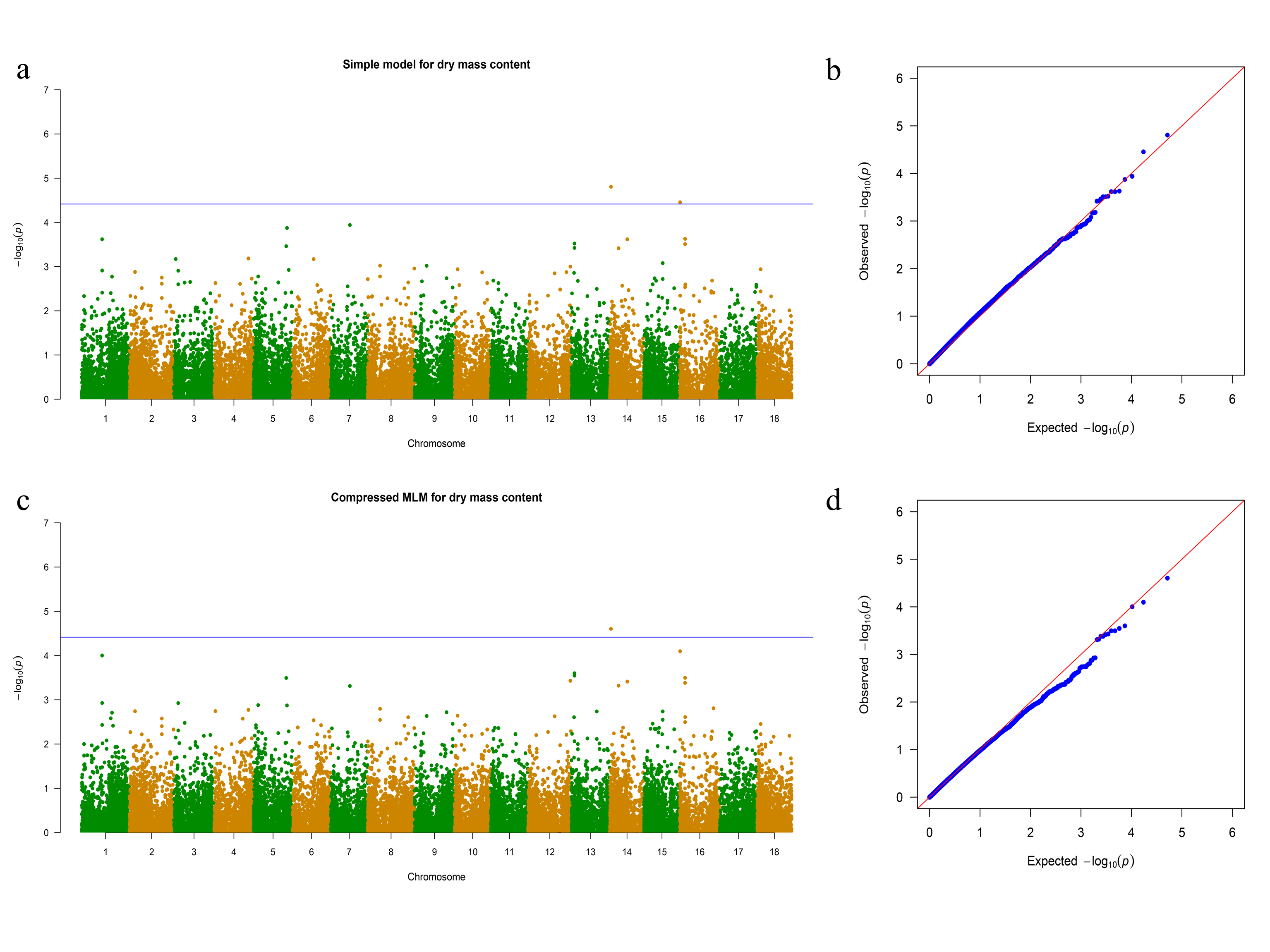

Supplement: FIGURE S4 — Genome-wide association analyses on dry mass content. (a) Manhattan plots of the simple model for dry mass content. Negative log10-transformed P values from a genome-wide scan are plotted against position on each of the 18 chromosomes. Genome-wide significance threshold is depicted as a blue horizontal dashed line. (b) Quantile–quantile plot of the simple model for dry mass content. (c) Manhattan plots of the compressed MLM for traits as in a. (d) Quantile–quantile plot of the compressed MLM for dry mass content. [file Image_4.TIF]

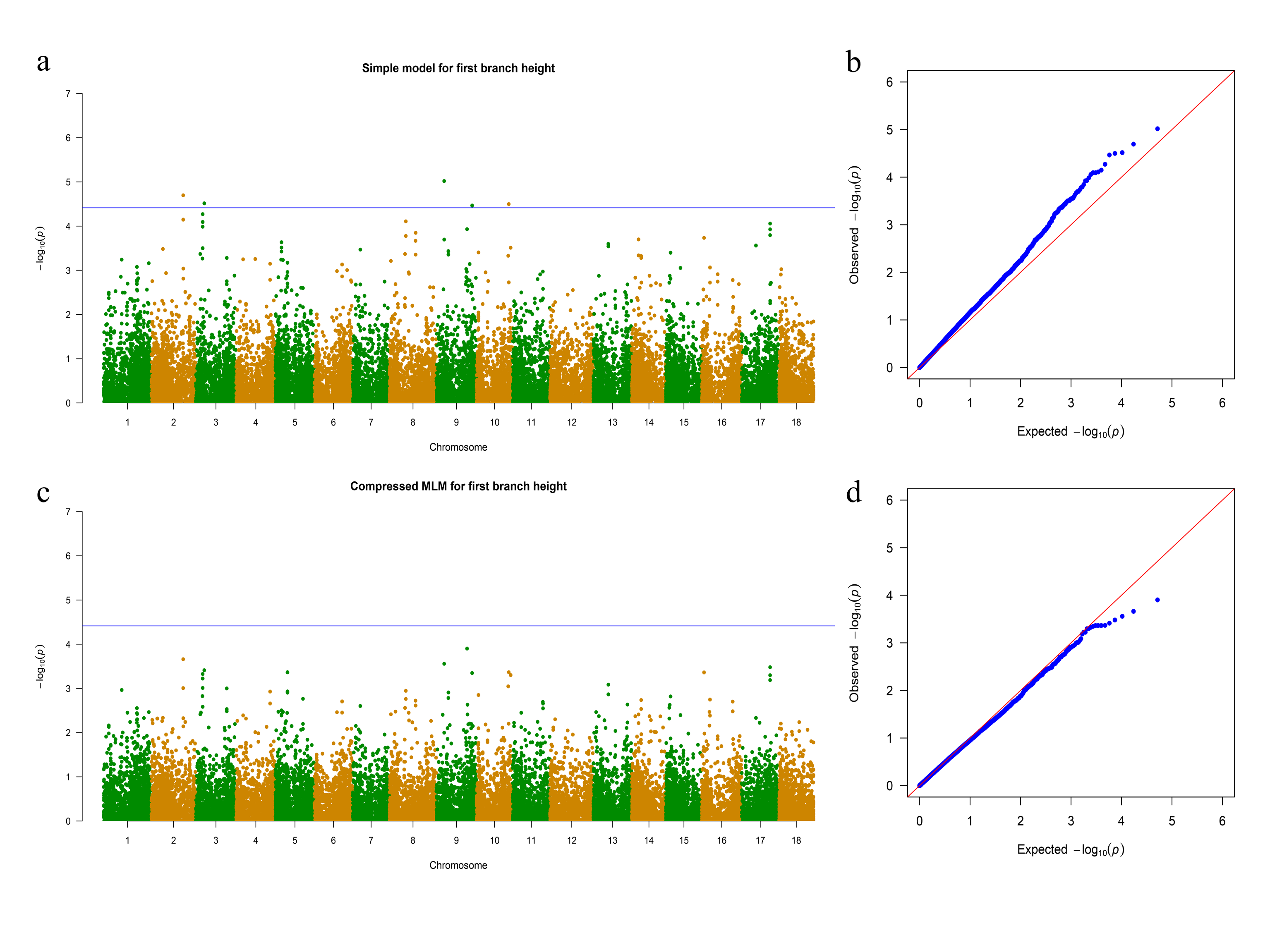

Supplement: FIGURE S5 — Genome-wide association analyses on first branch height. (a) Manhattan plots of the simple model for first branch height. Negative log10-transformed P values from a genome-wide scan are plotted against position on each of the 18 chromosomes. Genome-wide significance threshold is depicted as a blue horizontal dashed line. (b) Quantile–quantile plot of the simple model for first branch height. (c) Manhattan plots of the compressed MLM for traits as in a. (d) Quantile–quantile plot of the compressed MLM for first branch height. [file Image_5.TIF]

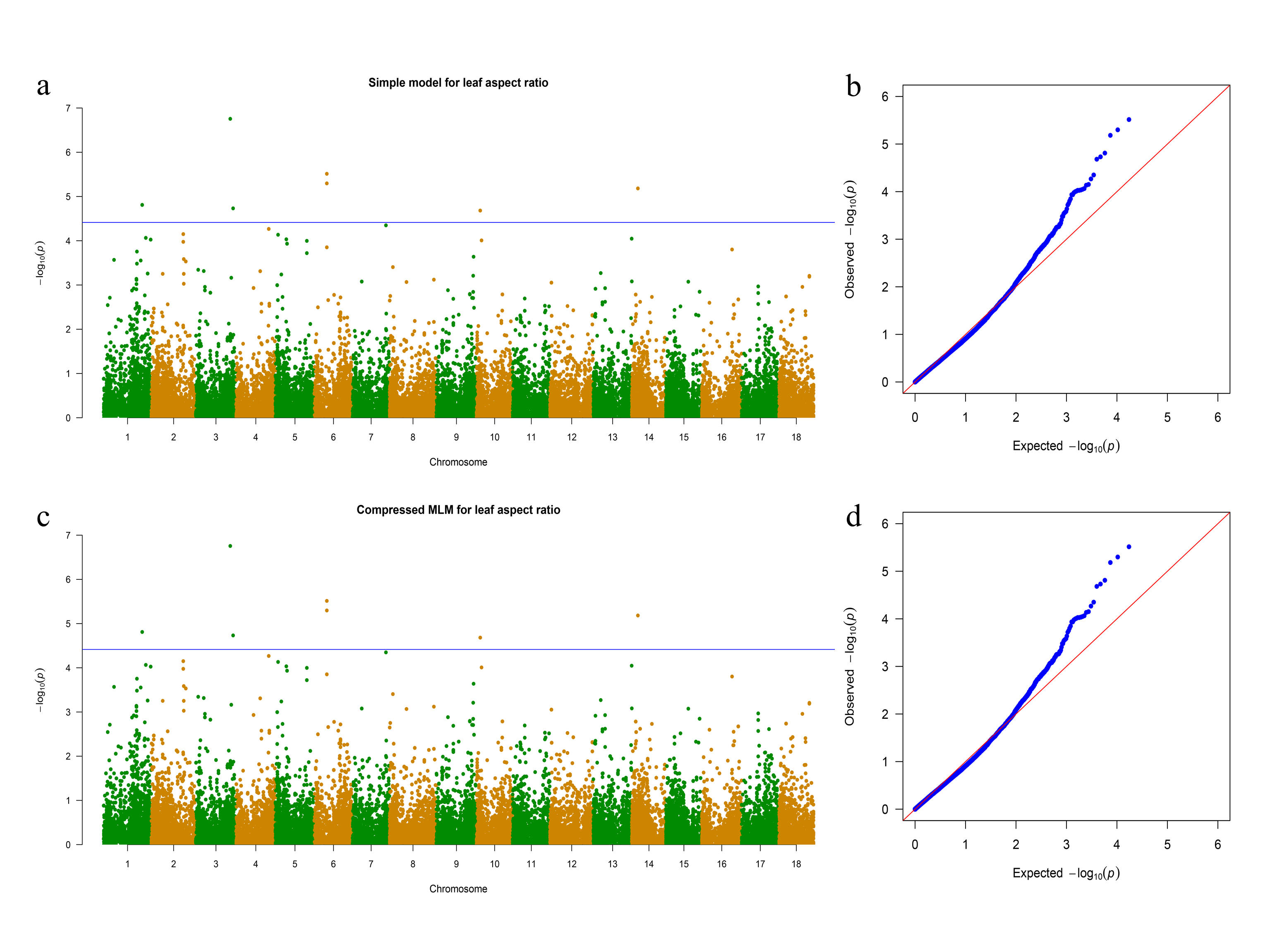

Supplement: FIGURE S6 — Genome-wide association analyses on leaf aspect ratio. (a) Manhattan plots of the simple model for leaf aspect ratio. Negative log10-transformed P values from a genome-wide scan are plotted against position on each of the 18 chromosomes. Genome-wide significance threshold is depicted as a blue horizontal dashed line. (b) Quantile–quantile plot of the simple model for leaf aspect ratio. (c) Manhattan plots of the compressed MLM for traits as in a. (d) Quantile–quantile plot of the compressed MLM for leaf aspect ratio. [file Image_6.TIF]

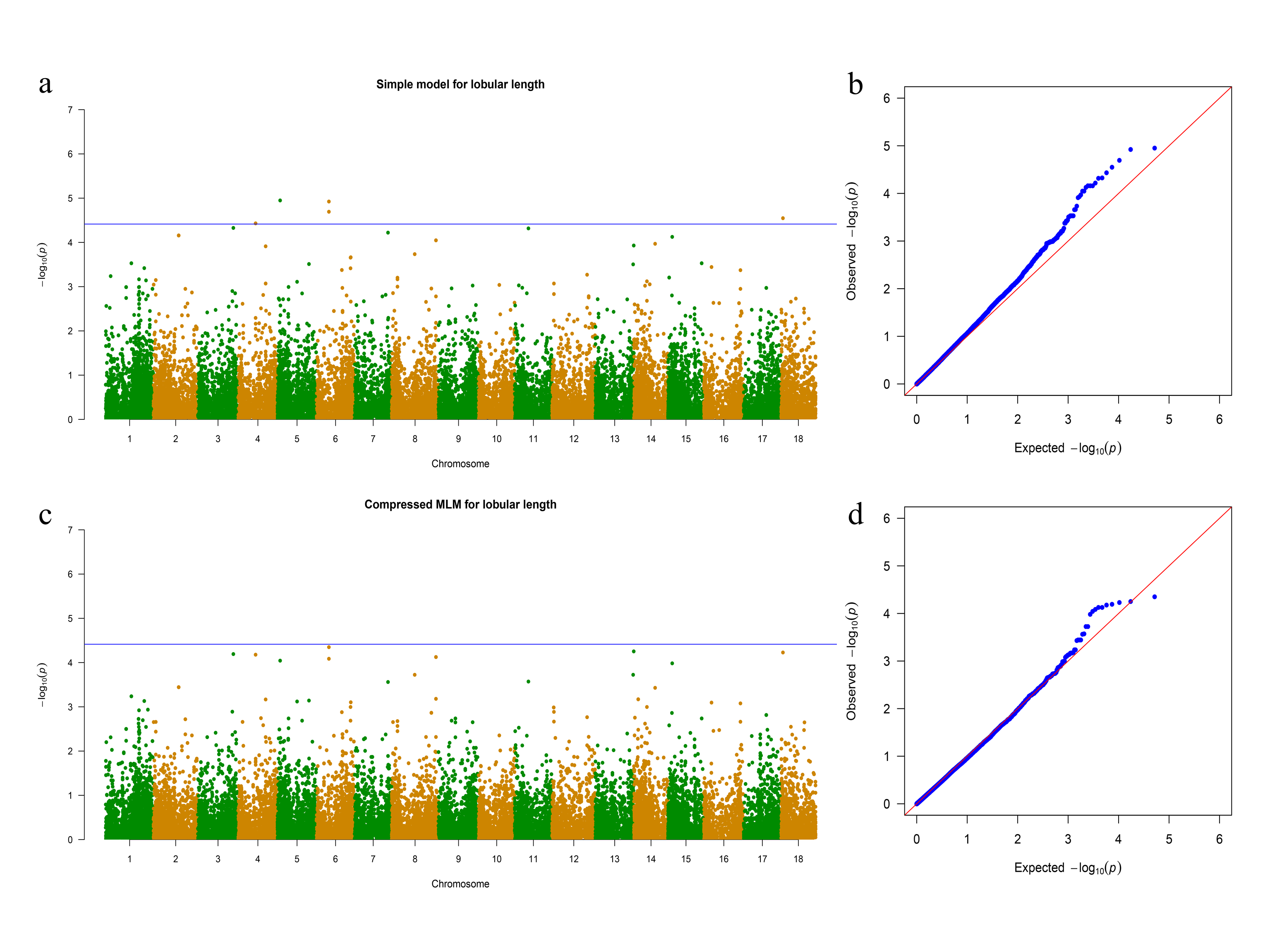

Supplement: FIGURE S7 — Genome-wide association analyses on lobular length. (a) Manhattan plots of the simple model for lobular length. Negative log10-transformed P values from a genome-wide scan are plotted against position on each of the 18 chromosomes. Genome-wide significance threshold is depicted as a blue horizontal dashed line. (b) Quantile–quantile plot of the simple model for lobular length. (c) Manhattan plots of the compressed MLM for traits as in a. (d) Quantile–quantile plot of the compressed MLM for lobular length. [file Image_7.TIF]

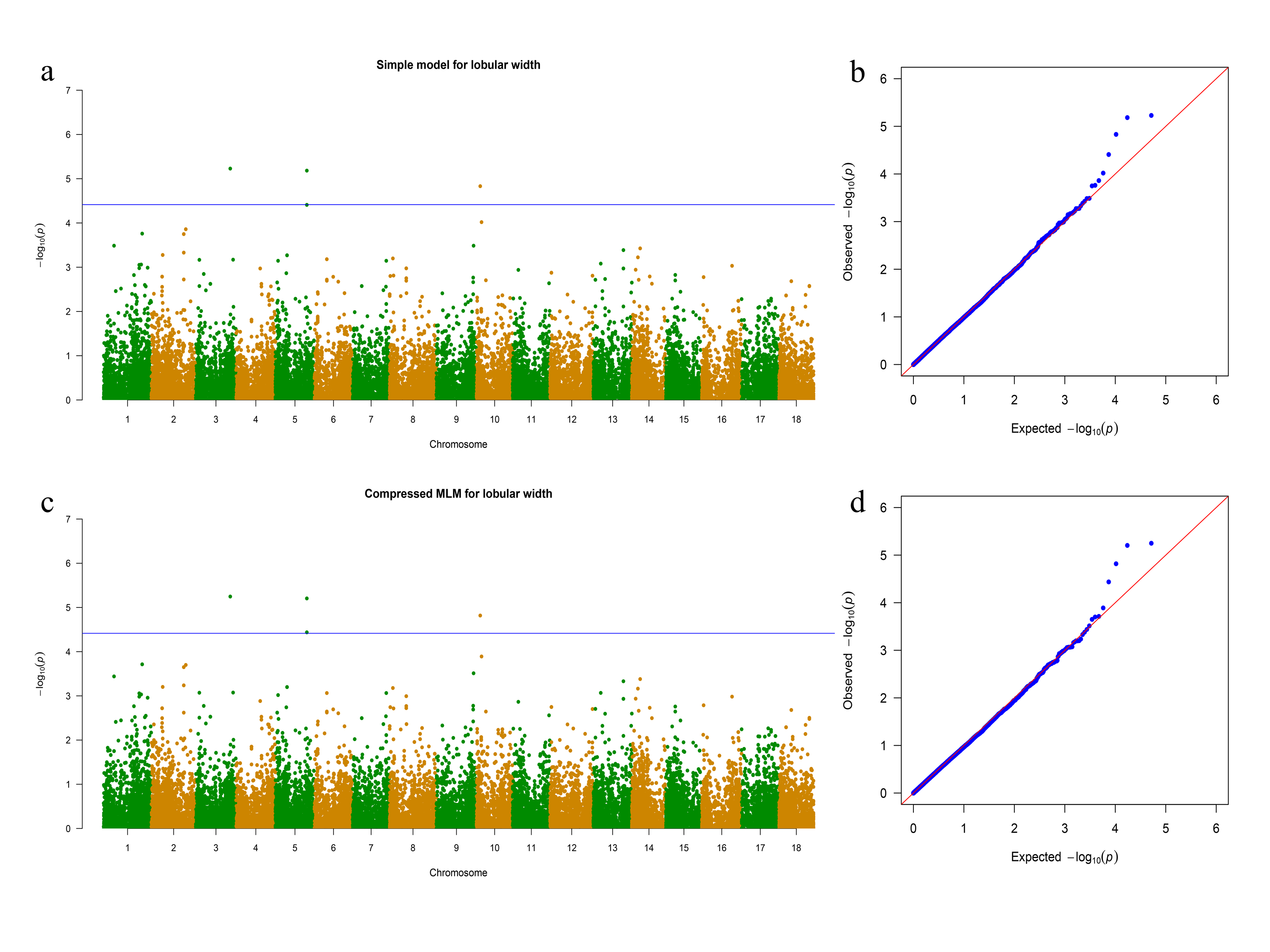

Supplement: FIGURE S8 — Genome-wide association analyses on lobular width. (a) Manhattan plots of the simple model for lobular width. Negative log10-transformed P values from a genome-wide scan are plotted against position on each of the 18 chromosomes. Genome-wide significance threshold is depicted as a blue horizontal dashed line. (b) Quantile–quantile plot of the simple model for lobular width. (c) Manhattan plots of the compressed MLM for lobular width as in a. (d) Quantile–quantile plot of the compressed MLM for lobular width. [file Image_8.TIF]

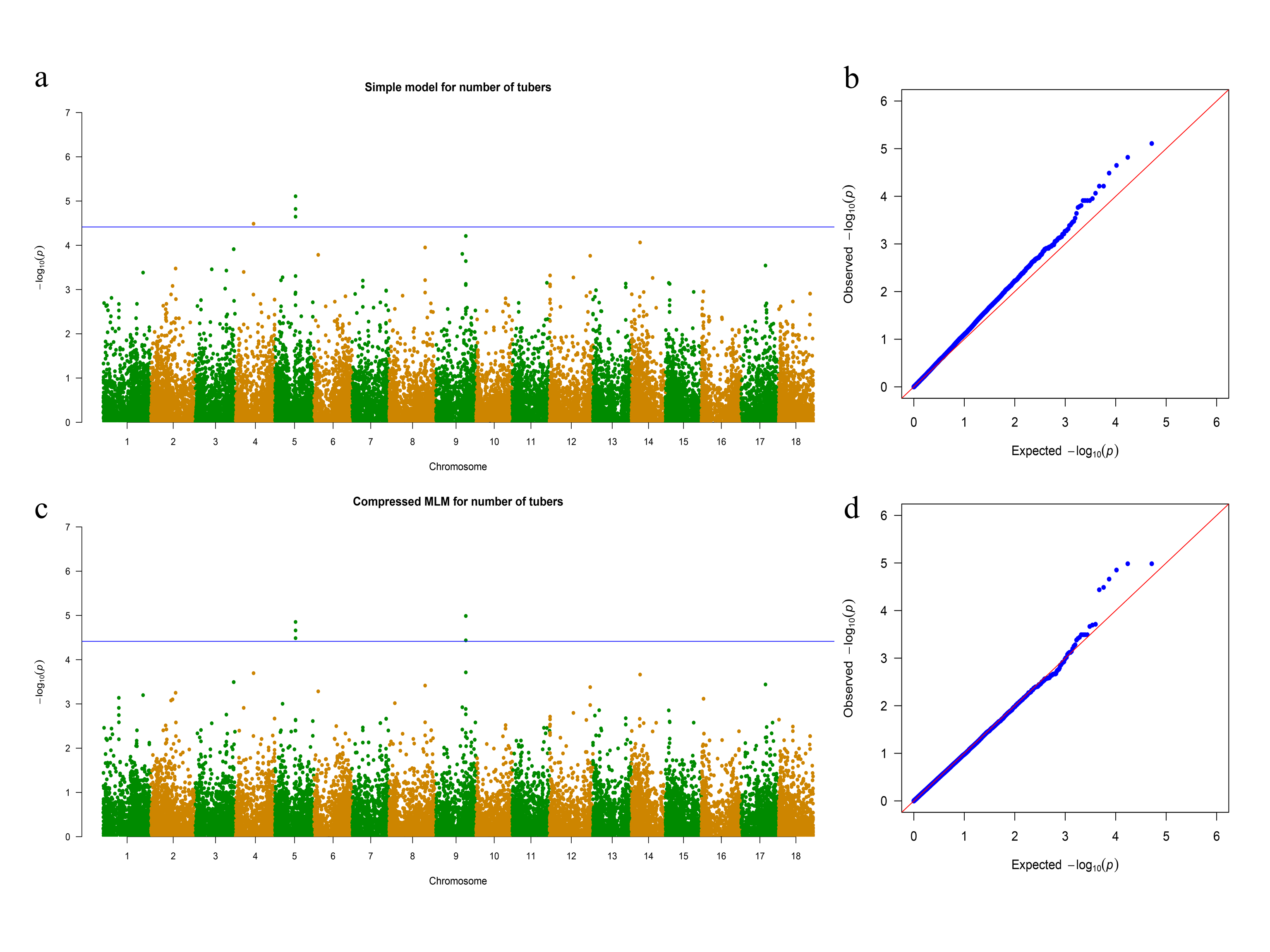

Supplement: FIGURE S9 — Genome-wide association analyses on the number of storage roots. (a) Manhattan plots of the simple model for the number of storage roots. Negative log10-transformed P values from a genome-wide scan are plotted against position on each of the 18 chromosomes. Genome-wide significance threshold is depicted as a blue horizontal dashed line. (b) Quantile–quantile plot of the simple model for traits. (c) Manhattan plots of the compressed MLM for the number of storage roots as in a. (d) Quantile–quantile plot of the compressed MLM for the number of storage roots. [file Image_9.TIF]

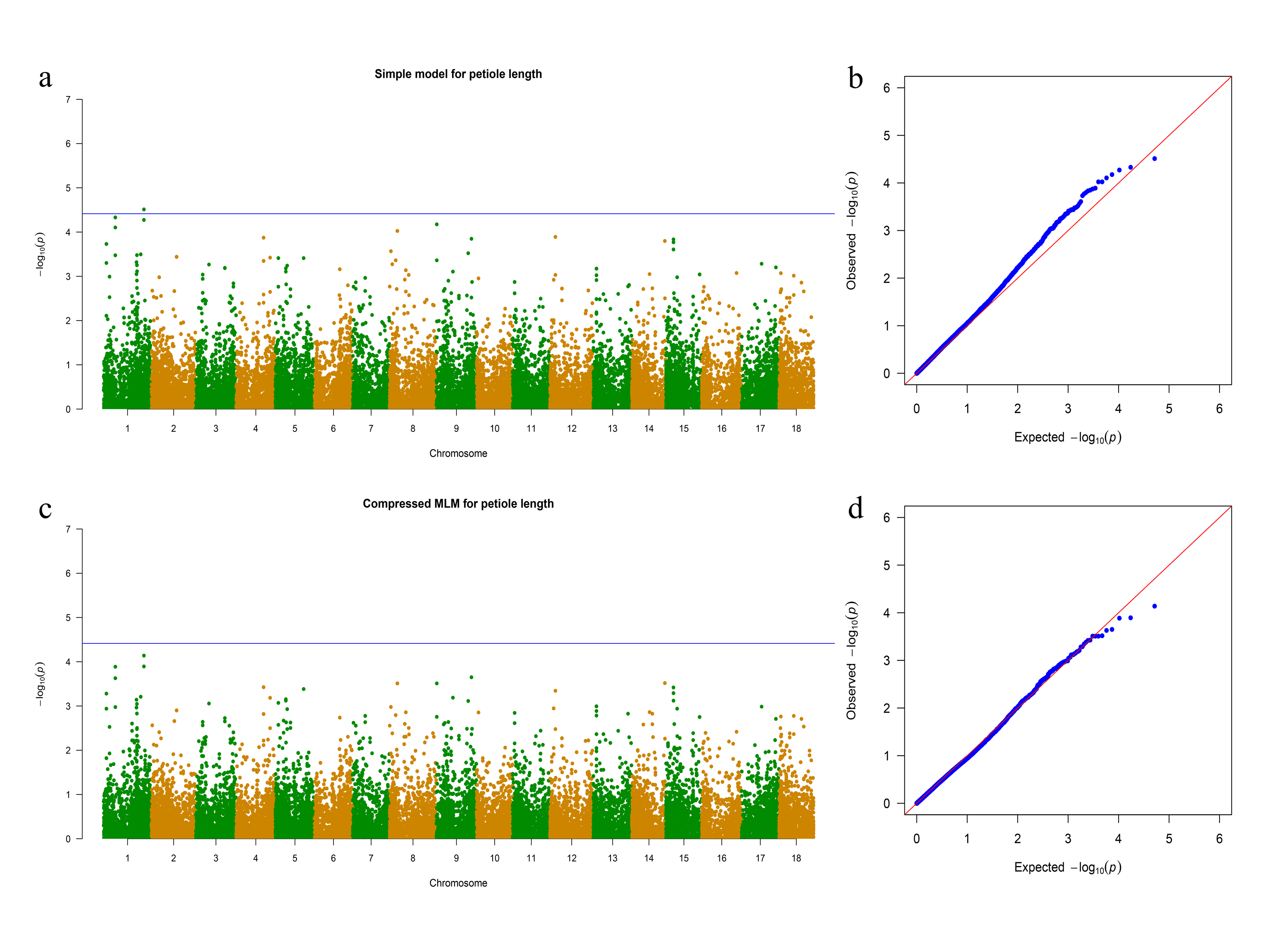

Supplement: FIGURE S10 — Genome-wide association analyses on petiole length. (a) Manhattan plots of the simple model for petiole length. Negative log10-transformed P values from a genome-wide scan are plotted against position on each of the 18 chromosomes. Genome-wide significance threshold is depicted as a blue horizontal dashed line. (b) Quantile–quantile plot of the simple model for traits. (c) Manhattan plots of the compressed MLM for petiole length as in a. (d) Quantile–quantile plot of the compressed MLM for petiole length. [file Image_10.TIF]

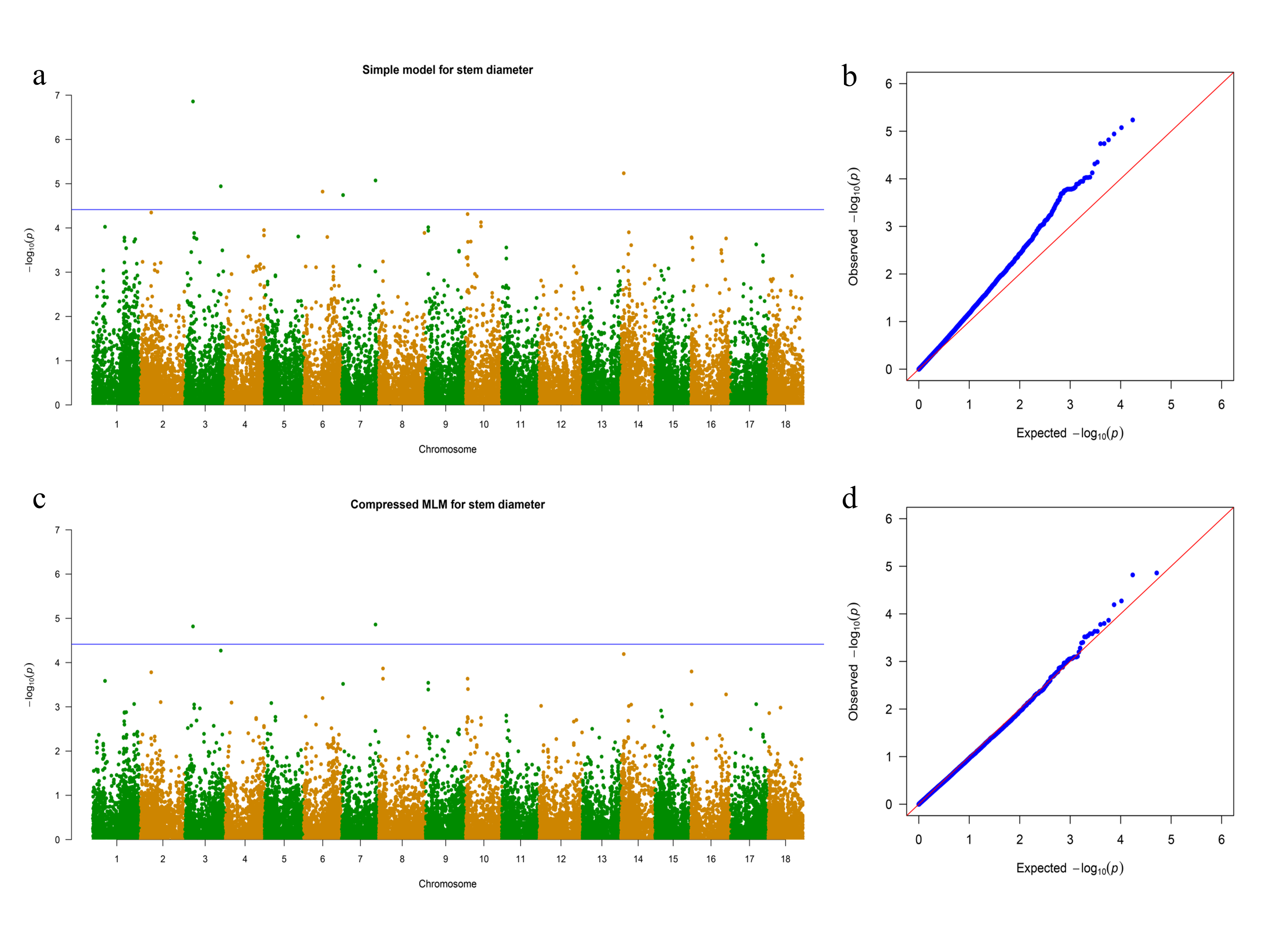

Supplement: FIGURE S11 — Genome-wide association analyses on stem diameter. (a) Manhattan plots of the simple model for stem diameter. Negative log10-transformed P values from a genome-wide scan are plotted against position on each of the 18 chromosomes. Genome-wide significance threshold is depicted as a blue horizontal dashed line. (b) Quantile–quantile plot of the simple model for traits. (c) Manhattan plots of the compressed MLM for stem diameter as in a. (d) Quantile–quantile plot of the compressed MLM for stem diameter. [file Image_11.TIF]

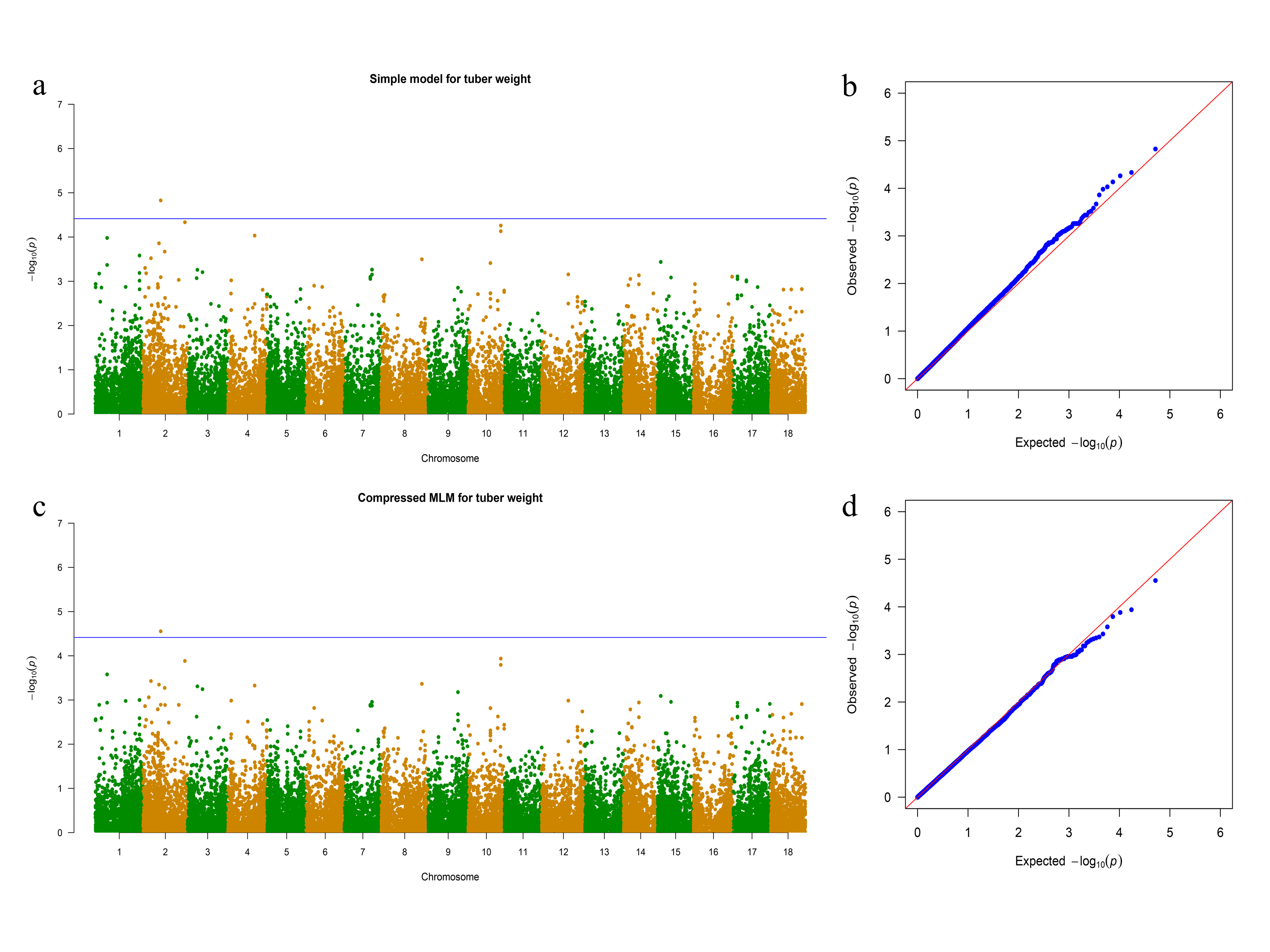

Supplement: FIGURE S12 — Genome-wide association analyses on storage roots weight. (a) Manhattan plots of the simple model for storage roots weight. Negative log10-transformed P values from a genome-wide scan are plotted against position on each of the 18 chromosomes. Genome-wide significance threshold is depicted as a blue horizontal dashed line. (b) Quantile–quantile plot of the simple model for storage roots weight. (c) Manhattan plots of the compressed MLM for traits as in a. (d) Quantile–quantile plot of the compressed MLM for storage roots weight. [file Image_12.TIF]

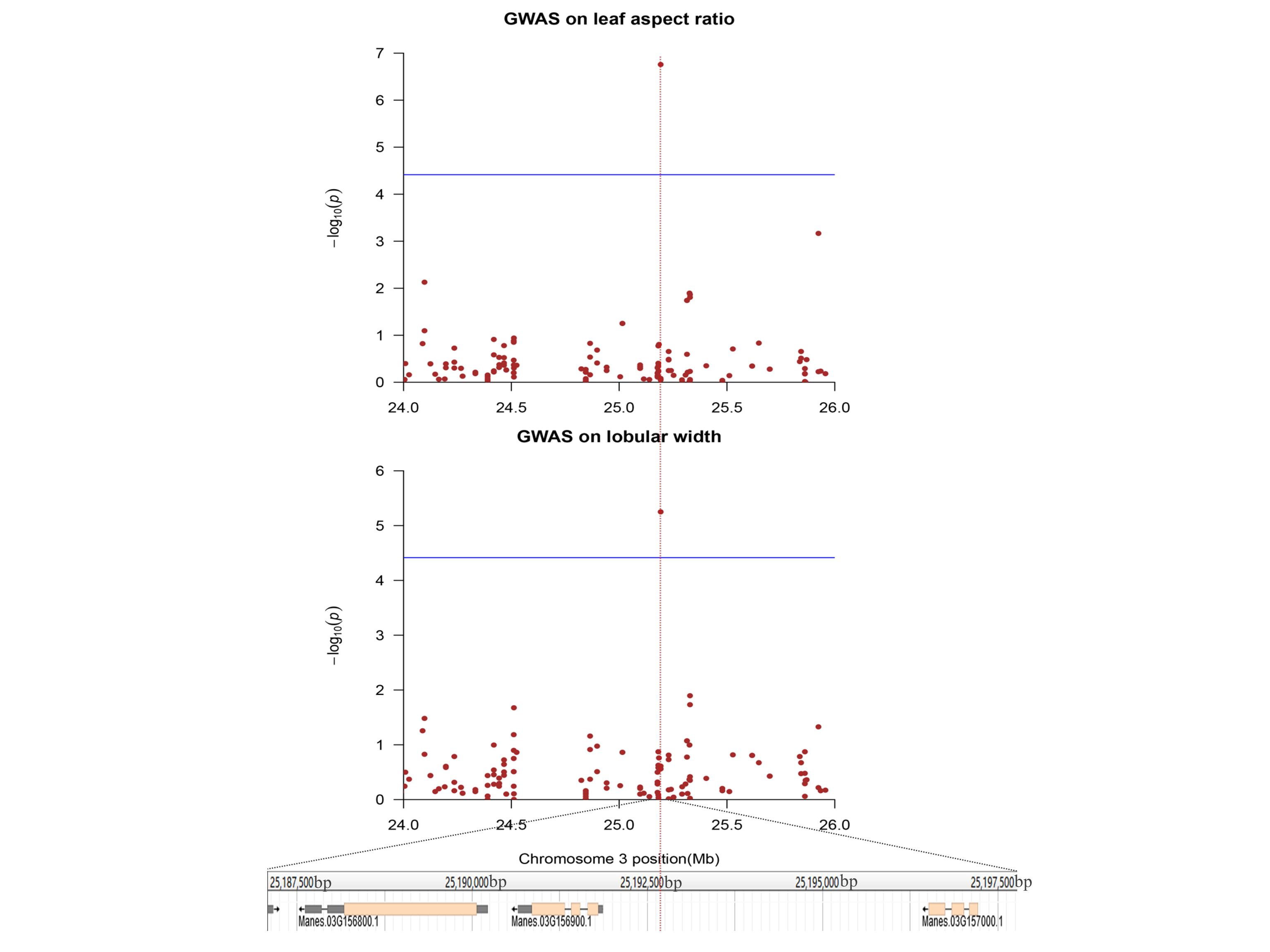

Supplement: FIGURE S13 — Genome-wide association analysis on leaf aspect ratio and lobular width in 158 cassava accessions using the SNPs detected on Chromosome 3. Genomic position (x axis) is plotted against its significance expressed as -log10 P value (y axis). Genomic position covers 1 Mb on either side of the peak SNP, as shown in a black dashed vertical line. Genome-wide significance threshold is depicted as a blue horizontal dashed line. Annotated candidate genes are indicated in pink boxes below the graph. [file Image_13.TIF]

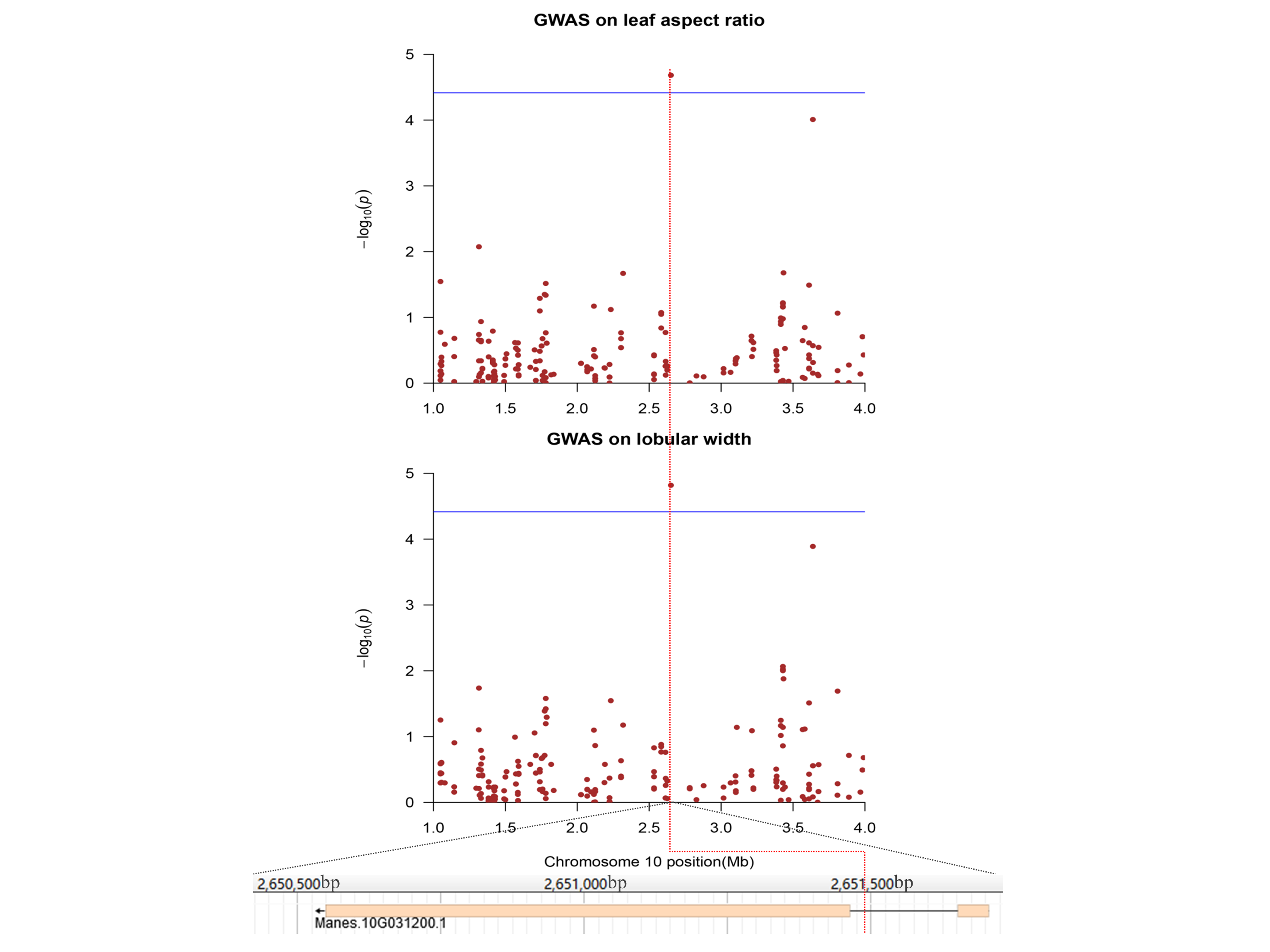

Supplement: FIGURE S14 — Genome-wide association analysis on leaf aspect ratio and lobular width in 158 cassava accessions using the SNPs detected on Chromosome 10. Genomic position (x axis) is plotted against its significance expressed as -log10 P value (y axis). Genomic position covers 1 Mb on either side of the peak SNP, as shown in a black dashed vertical line. Genome-wide significance threshold is depicted as a blue horizontal dashed line. Annotated candidate genes are indicated in pink boxes below the graph. [file Image_14.TIF]

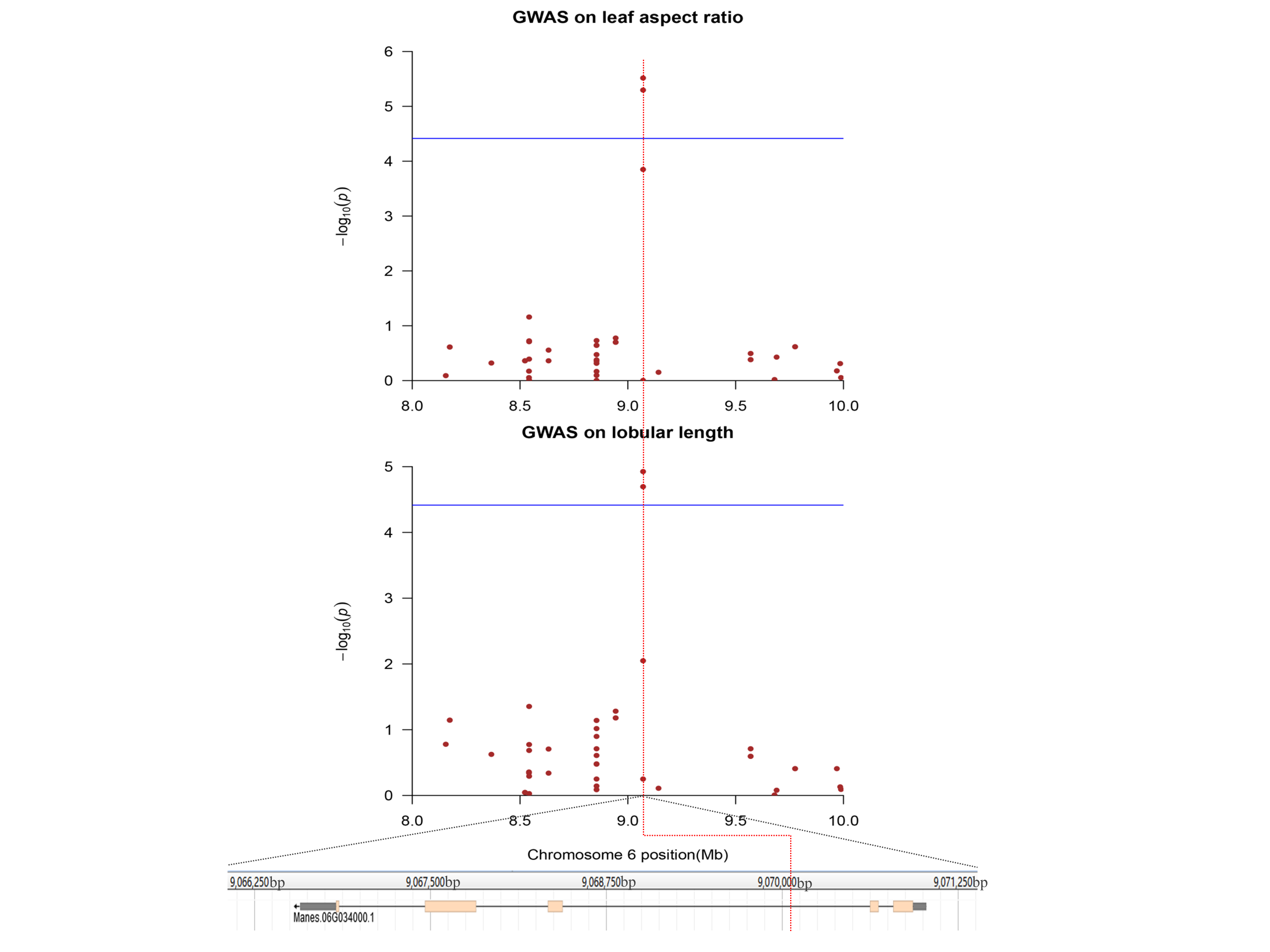

Supplement: FIGURE S15 — Genome-wide association analysis on leaf aspect ratio and lobular length in 158 cassava accessions using the SNPs detected on Chromosome 6. Genomic position (x axis) is plotted against its significance expressed as -log10 P value (y axis). Genomic position covers 1 Mb on either side of the peak SNP, as shown in a black dashed vertical line. Genome-wide significance threshold is depicted in blue horizontal dashed line. Annotated candidate genes are indicated in pink boxes below the graph. [file Image_15.TIF]
